# Supplementary material for: A critical region of A20 unveiled by missense TNFAIP3 variations that lead to autoinflammation
Source: eLife. 2023 Jun 21;12:e81280. doi: 10.7554/eLife.81280 (PMC10284599; doi:10.7554/eLife.81280)
Supplement: Figure 3—source data 1. — Uncropped western blot images of A20 and GAPDH protein expression in PBMCs of the patients and healthy individuals. [file elife-81280-fig3-data1.pdf]

Figure 3A

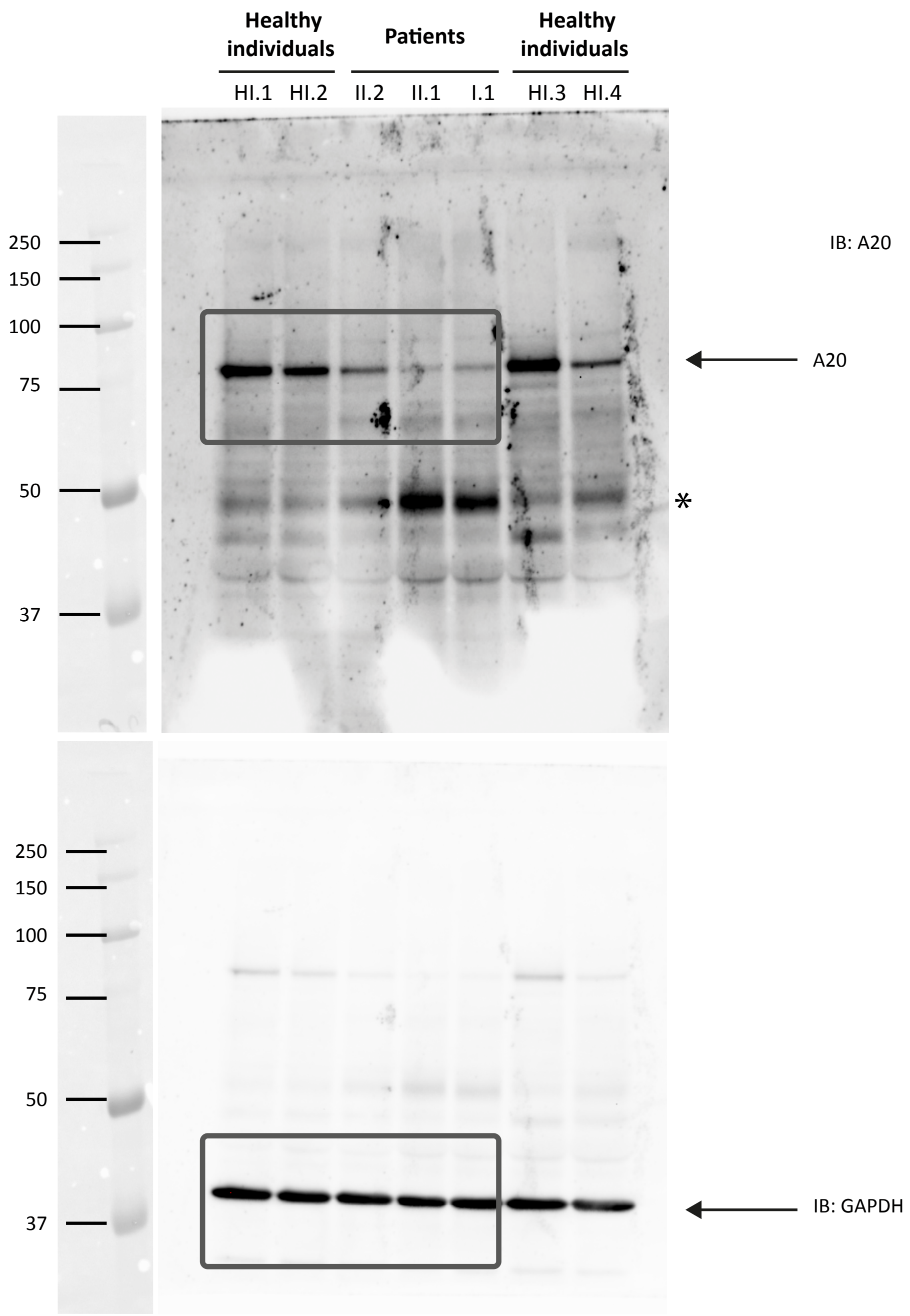

*: band that is not specific as seen on other WB from PBMC protein extracts of the patients and other healthy individuals
