# Supplementary figures and images for: A critical region of A20 unveiled by missense TNFAIP3 variations that lead to autoinflammation
Source: eLife. 2023 Jun 21;12:e81280. doi: 10.7554/eLife.81280 (PMC10284599; doi:10.7554/eLife.81280)

Figure 3B

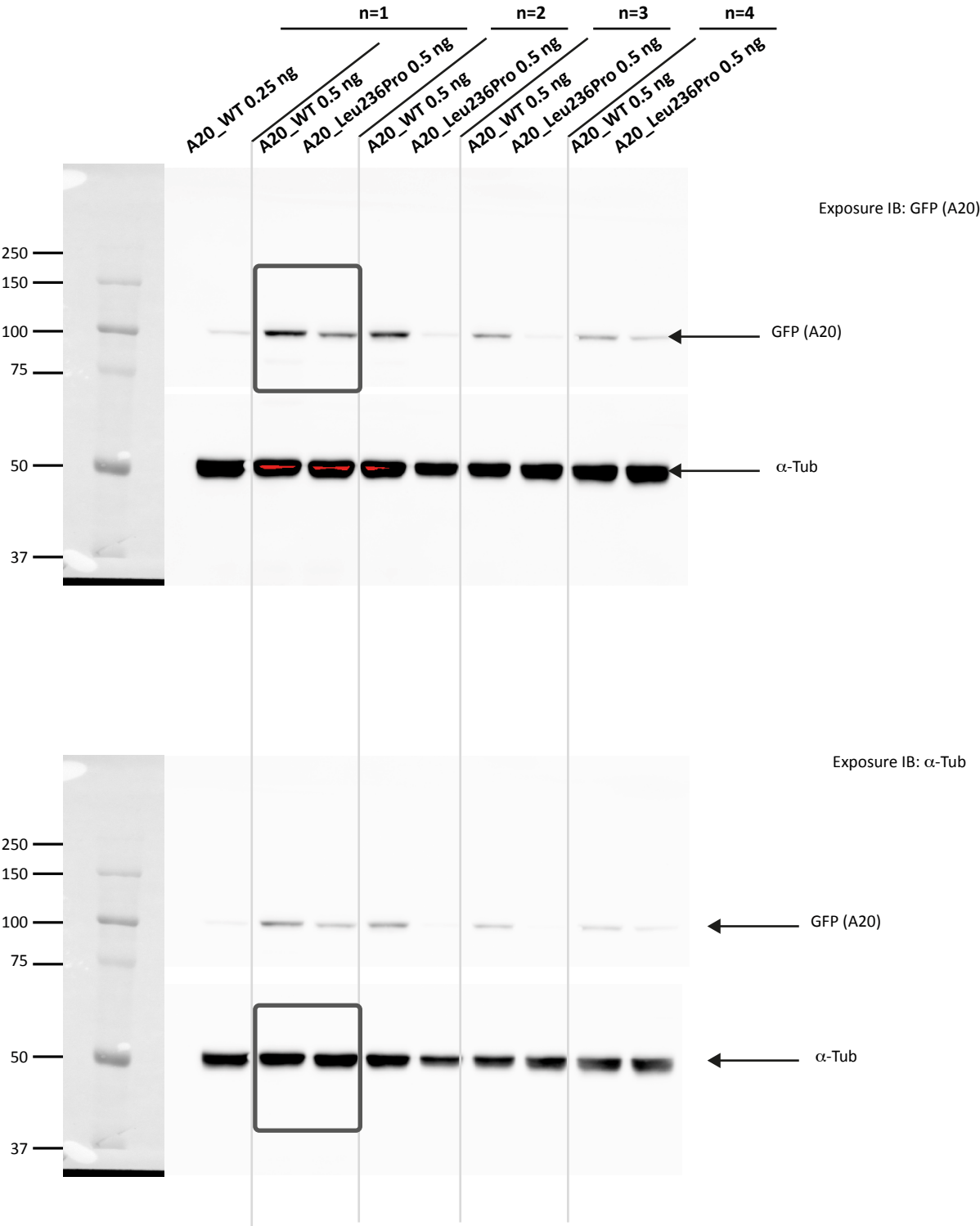

Supplement: Figure 3—source data 2. — Uncropped western blot images of GFP-A20 and α-tubulin protein expression (n=4). [file elife-81280-fig3-data2.pdf]

Figure 3C

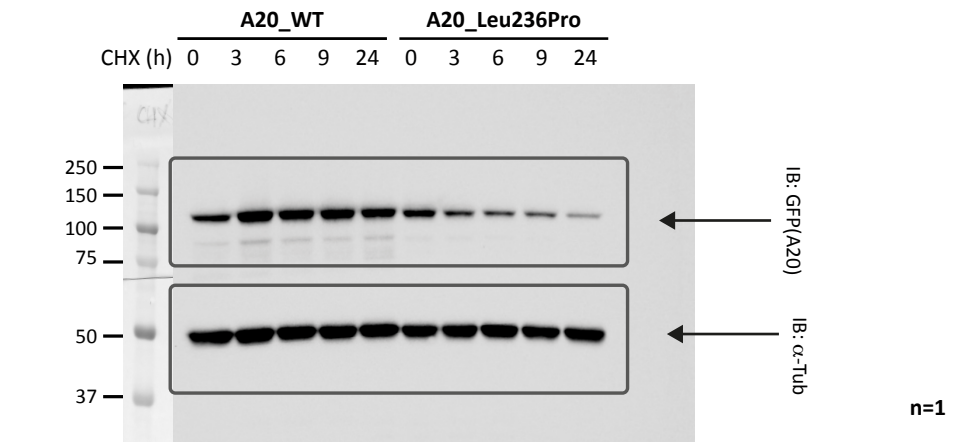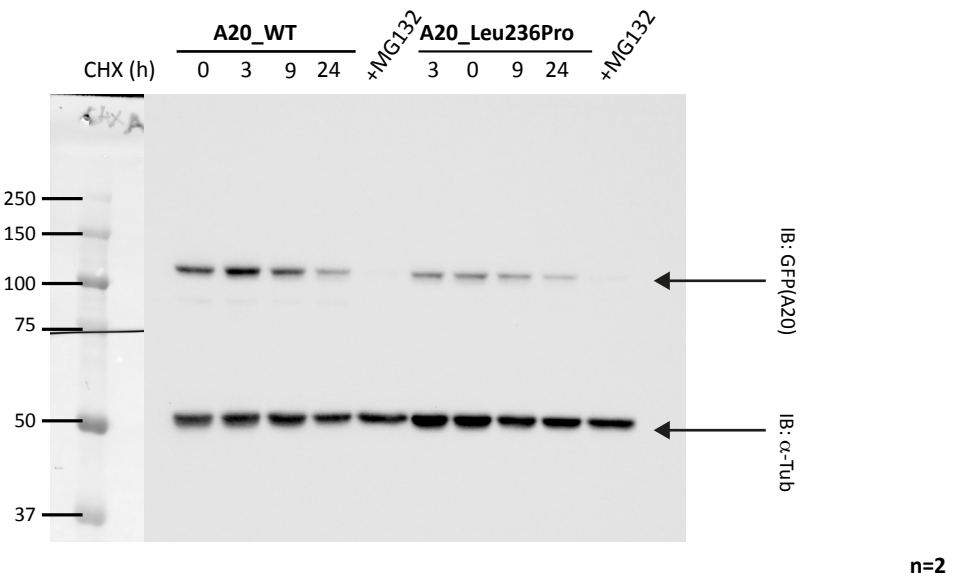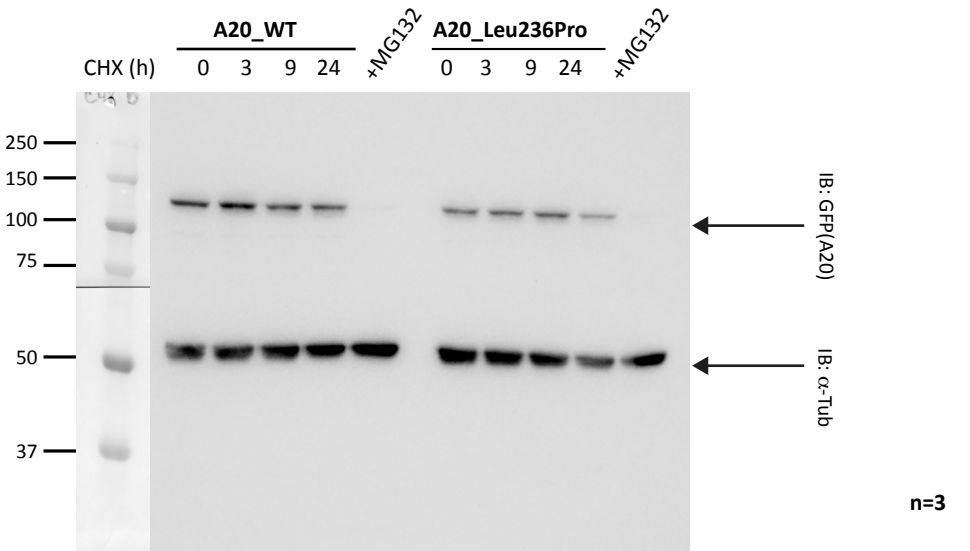

Supplement: Figure 3—source data 3. — Uncropped western blot images of GFP-A20 and α-tubulin protein expression (n=3). [file elife-81280-fig3-data3.pdf]

Figure 3D

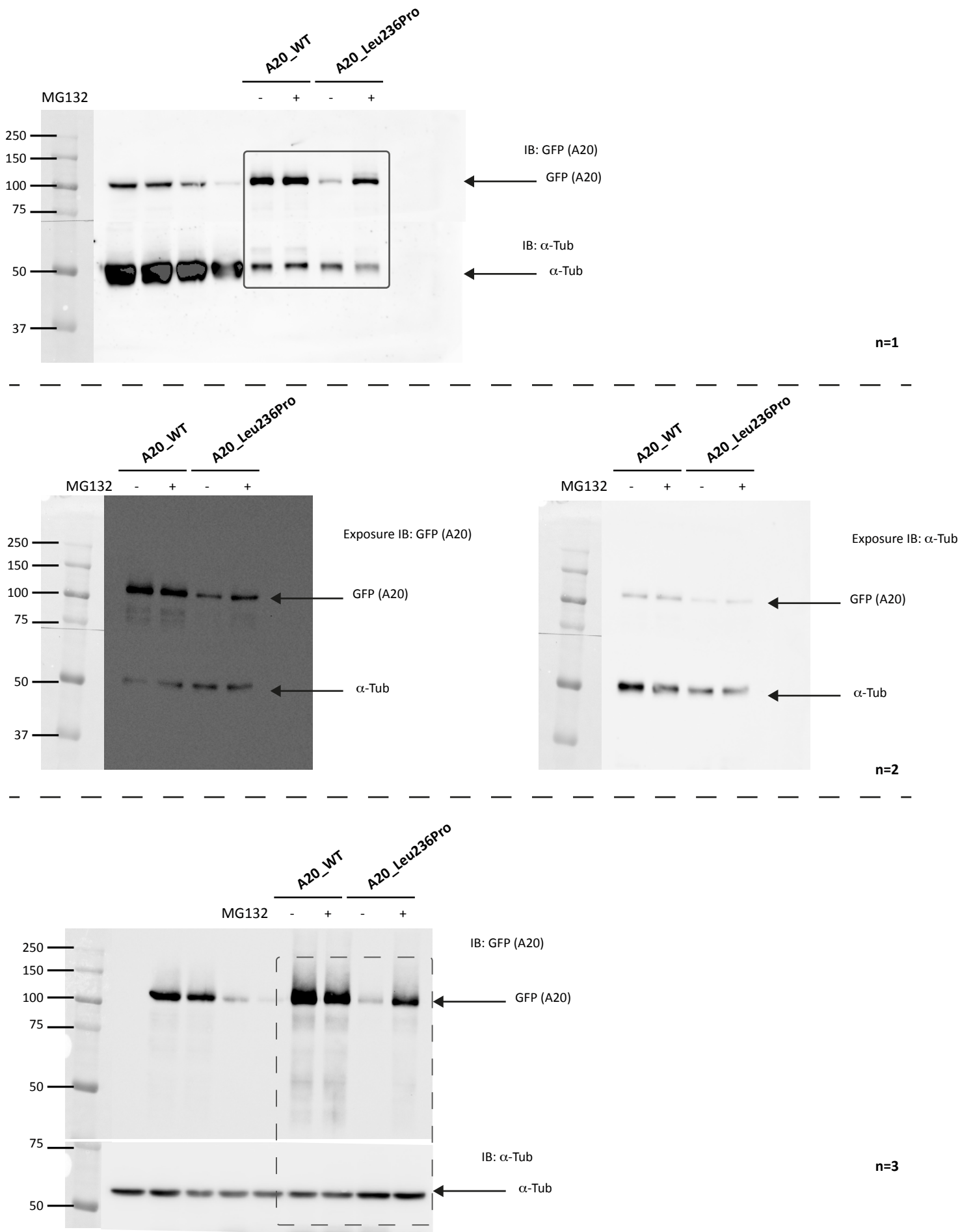

Supplement: Figure 3—source data 4. — Uncropped western blot images of GFP-A20 and α-tubulin protein expression (n=3). [file elife-81280-fig3-data4.pdf]
