# Supplementary material for: A critical region of A20 unveiled by missense TNFAIP3 variations that lead to autoinflammation
Source: eLife. 2023 Jun 21;12:e81280. doi: 10.7554/eLife.81280 (PMC10284599; doi:10.7554/eLife.81280)
Supplement: Figure 3—source data 5. — Uncropped western blot images of Flag immunoprecipitated samples (HA-ubiquitin and Flag-TRAF6) and crude extracts: GFP-A20, Flag-TRAF6, and GAPDH (n=3). [file elife-81280-fig3-data5.pdf]

Figure 3F n=1

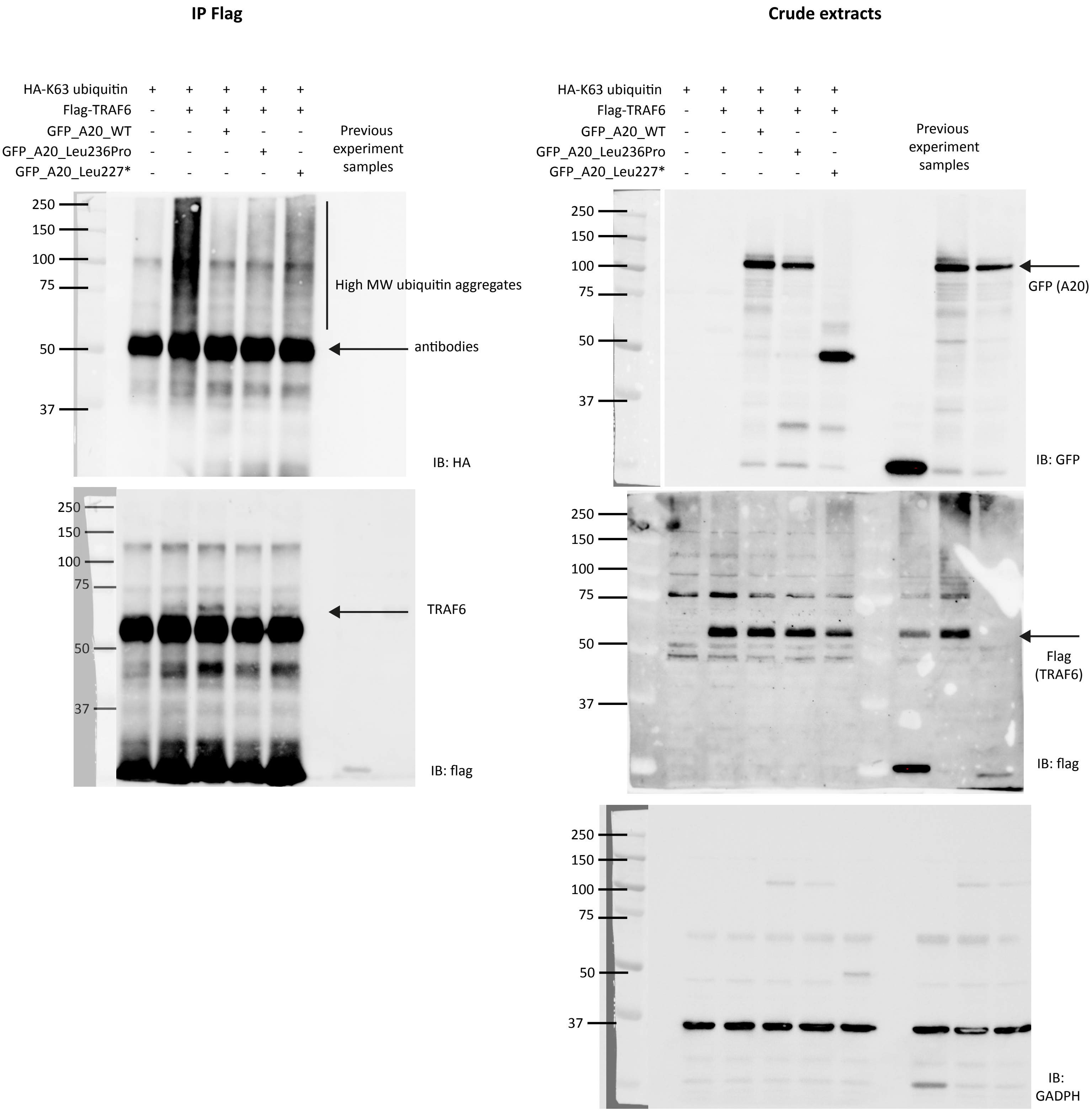
IP Flag
Crude extracts
HA-K63 ubiquitin + + + + +
Flag-TRAF6 - + + + +
GFP_A20_WT - - + - -
GFP_A20_Leu236Pro - - - + -
GFP_A20_Leu227* - - - - +
Previous experiment samples
250
150
100
75
50
37
High MW ubiquitin aggregates
antibodies
IB: HA
TRAF6
IB: flag
GFP (A20)
IB: GFP
Flag (TRAF6)
IB: flag
IB: GADPH

Figure 3F n=2

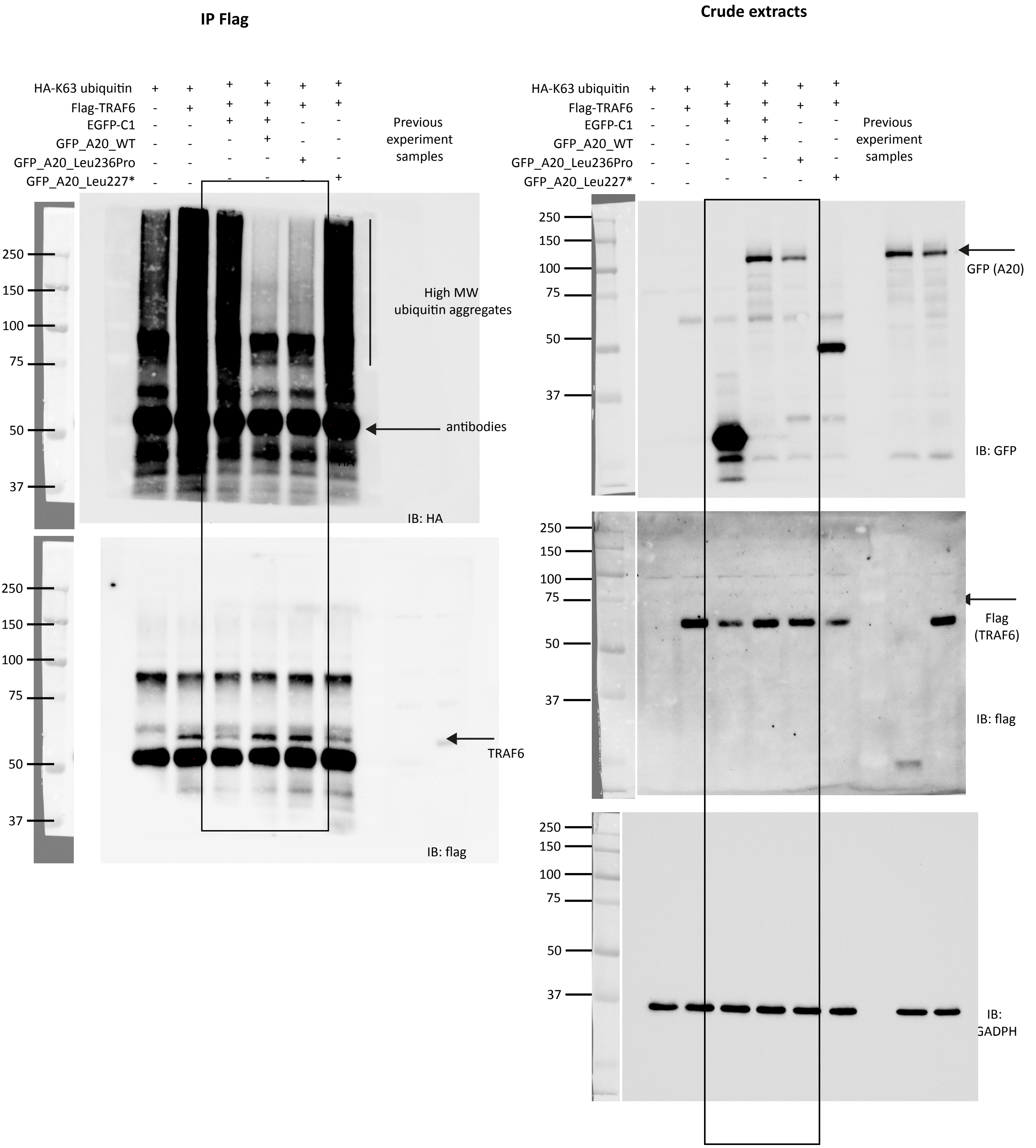
IP Flag
Crude extracts
HA-K63 ubiquitin
Flag-TRAF6
EGFP-C1
GFP_A20_WT
GFP_A20_Leu236Pro
GFP_A20_Leu227*
Previous experiment samples
250
150
100
75
50
37
High MW ubiquitin aggregates
antibodies
IB: HA
TRAF6
IB: flag
GFP (A20)
IB: GFP
Flag (TRAF6)
IB: flag
IB: GADPH

Figure 3F n=3

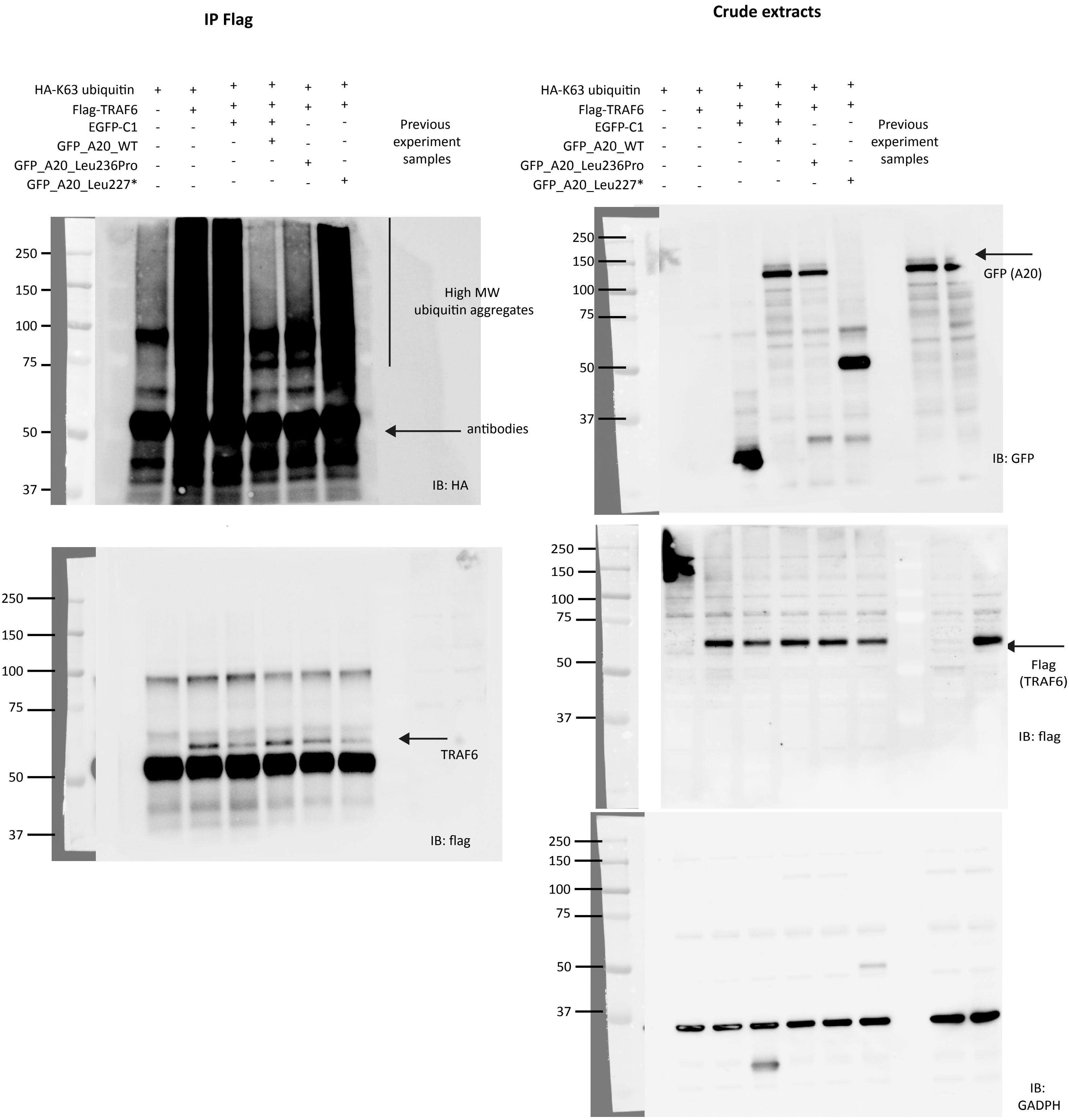
IP Flag
Crude extracts
HA-K63 ubiquitin
Flag-TRAF6
EGFP-C1
GFP_A20_WT
GFP_A20_Leu236Pro
GFP_A20_Leu227*
Previous experiment samples
250
150
100
75
50
37
High MW ubiquitin aggregates
antibodies
IB: HA
TRAF6
IB: flag
GFP (A20)
IB: GFP
Flag (TRAF6)
IB: flag
IB: GADPH
