# Supplementary material for: A critical region of A20 unveiled by missense TNFAIP3 variations that lead to autoinflammation
Source: eLife. 2023 Jun 21;12:e81280. doi: 10.7554/eLife.81280 (PMC10284599; doi:10.7554/eLife.81280)
Supplement: Figure 4—source data 1. [file elife-81280-fig4-data1.pdf]

Results <Default Table>

FCS Key 1	FCS Key 2	Gate	Region	Count	%Gated	X Median	X Mean	Error Message
HEK NT	no trt	Ungated	A	20281	60.09	333.76	364.11	
HEK NT	no trt	A	B	59	0.29	32.78	80.43	
HEK EV	DMSO	Ungated	A	20442	66.14	345.99	372.32	
HEK EV	DMSO	A	B	2	0.01	9.14	407.49	
HEK EV	MG132 invivo	Ungated	A	20324	66.00	365.17	398.57	
HEK EV	MG132 invivo	A	B	2	0.01	8.82	23.68	
HEK EGFP	DMSO	Ungated	A	20320	60.16	361.90	395.20	
HEK EGFP	DMSO	A	B	621	3.06	105.54	547.22	
HEK EGFP	MG132 invivo	Ungated	A	20356	63.05	342.89	379.11	
HEK EGFP	MG132 invivo	A	B	5723	28.11	86.60	495.77	
HEK A20 wt	DMSO	Ungated	A	20337	66.03	339.82	373.60	
HEK A20 wt	DMSO	A	B	7941	39.05	41.42	233.82	
HEK A20 wt	MG132 invivo	Ungated	A	20372	60.69	339.82	375.11	
HEK A20 wt	MG132 invivo	A	B	7427	36.46	42.17	239.34	
HEK A20 L236P	DMSO	Ungated	A	20413	59.81	316.23	351.04	
HEK A20 L236P	DMSO	A	B	6363	31.17	33.38	129.95	
HEK A20 L236P	MG132 invivo	Ungated	A	20511	45.14	313.40	345.50	
HEK A20 L236P	MG132 invivo	A	B	6381	31.11	47.40	186.91	
HEK A20 N102S	DMSO	Ungated	A	20608	62.42	313.40	344.84	
HEK A20 N102S	DMSO	A	B	7971	38.68	42.55	238.46	
HEK A20 N102S	MG132 invivo	Ungated	A	20422	56.46	330.77	364.94	
HEK A20 N102S	MG132 invivo	A	B	7292	35.71	40.32	210.86	
HEK A20 F127C	DMSO	Ungated	A	20437	51.84	324.88	360.72	
HEK A20 F127C	DMSO	A	B	7042	34.46	39.60	203.55	
HEK A20 F127C	MG132 invivo	Ungated	A	20502	56.35	336.78	369.13	
HEK A20 F127C	MG132 invivo	A	B	7064	34.46	38.89	205.07	
HEK A20 L275P	DMSO	Ungated	A	20291	63.47	327.81	362.52	
HEK A20 L275P	DMSO	A	B	6944	34.22	38.89	163.08	
HEK A20 L275P	MG132 invivo	Ungated	A	20233	56.41	361.90	394.65	
HEK A20 L275P	MG132 invivo	A	B	6811	33.66	55.23	203.61	
HEK A20 L277	DMSO	Ungated	A	20326	65.97	330.77	366.91	
HEK A20 L277	DMSO	A	B	5508	27.10	31.62	57.52	
HEK A20 L277	MG132 invivo	Ungated	A	20278	56.96	345.99	380.09	
HEK A20 L277	MG132 invivo	A	B	7679	37.87	54.74	119.21	

Overlay 1

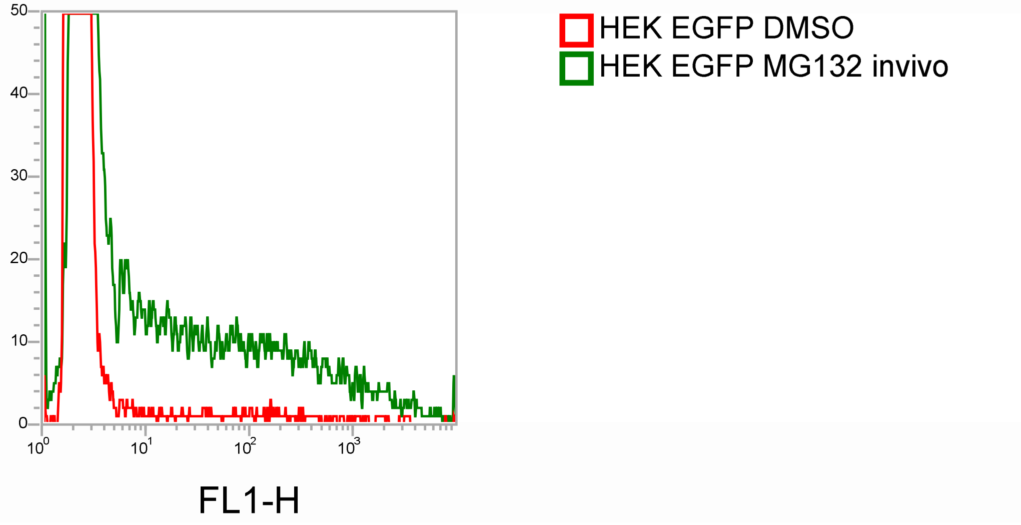

Overlay 2

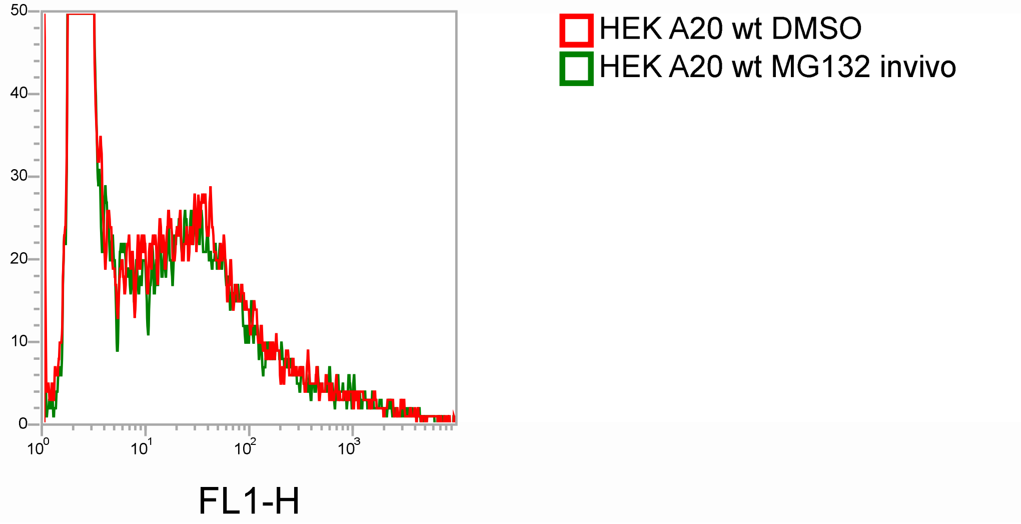

Overlay 3

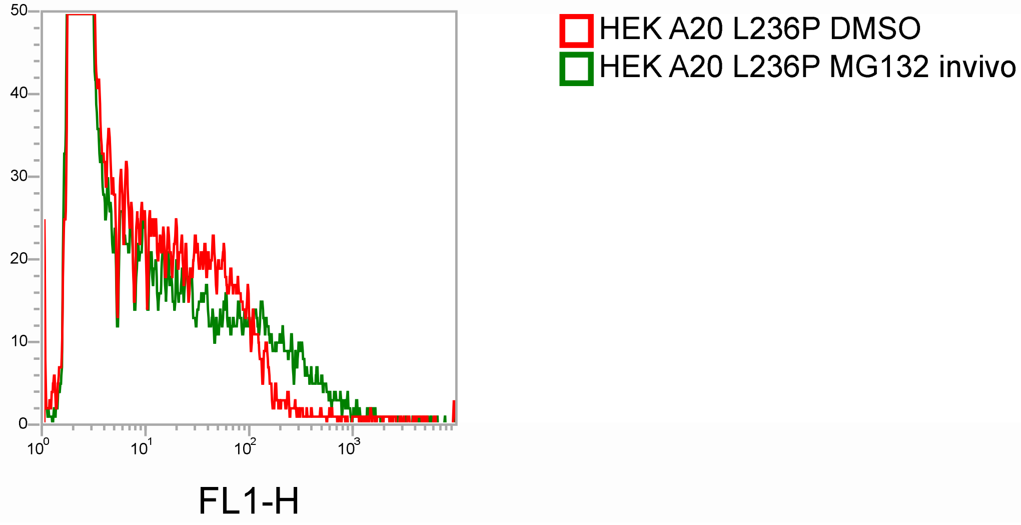

Overlay 4

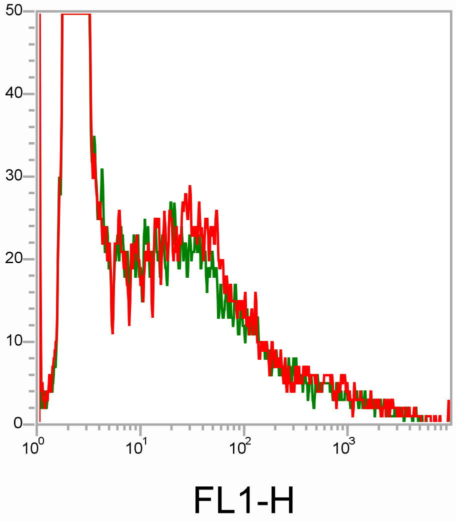

HEK A20 N102S DMSO
HEK A20 N102S MG132 invivo

Overlay 5

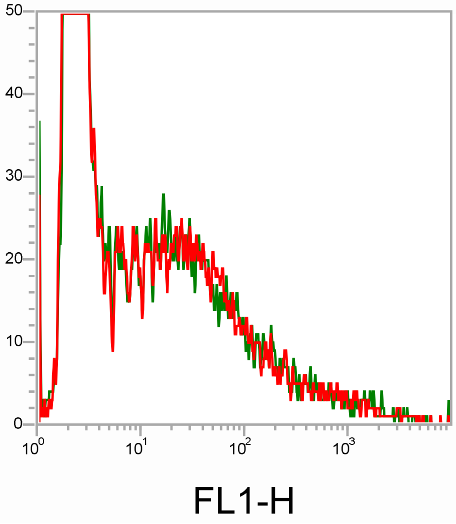

HEK A20 F127C DMSO
HEK A20 F127C MG132 invivo

Overlay 6

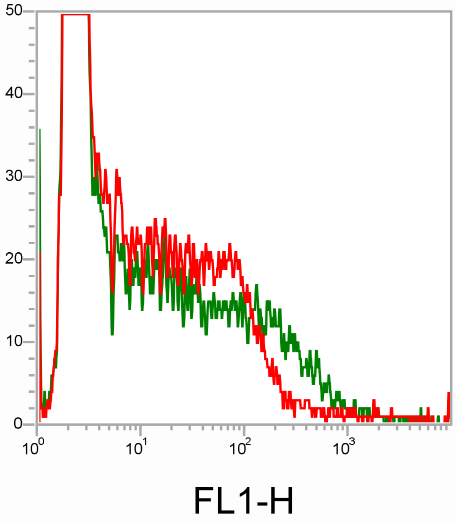

HEK A20 L275P DMSO
HEK A20 L275P MG132 invivo

Overlay 7

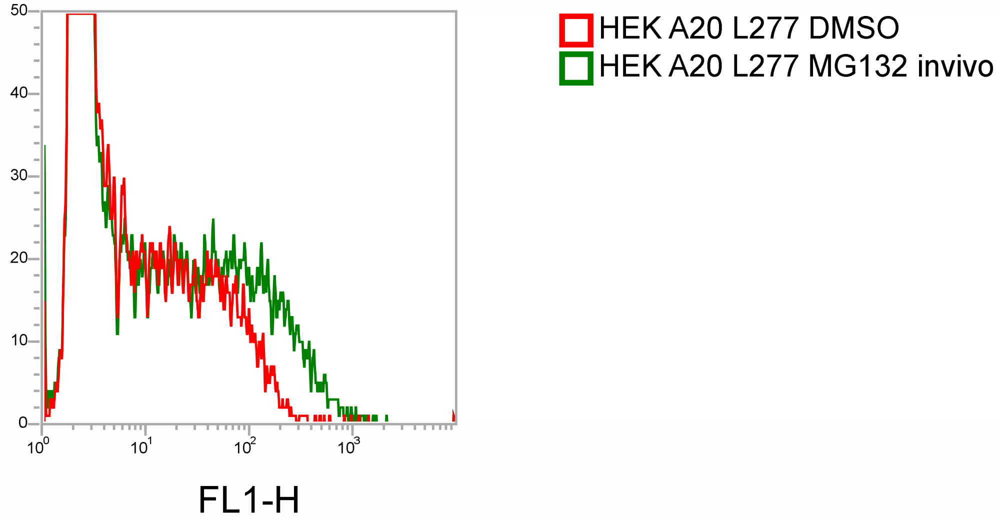

HEK NT no trt
(Ungated) 1

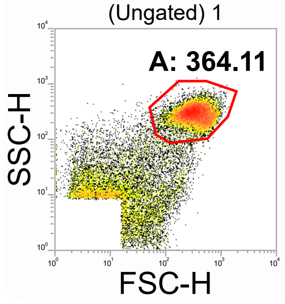

FCS Key 1	FCS Key 2	Gate	Region	Count	%Gated	X Median	X Mean	Error Message
HEK NT	no trt	Ungated	A	20281	60.09	333.76	364.11	

HEK NT no trt
(A) 2

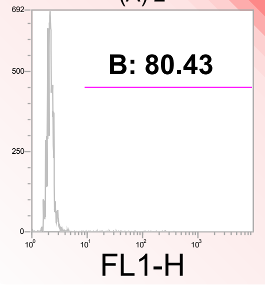

FCS Key 1	FCS Key 2	Gate	Region	Count	%Gated	X Median	X Mean	Error Message
HEK NT	no trt	A	B	59	0.29	32.78	80.43	

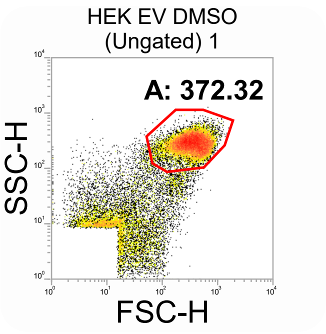

FCS Key 1	FCS Key 2	Gate	Region	Count	%Gated	X Median	X Mean	Error Message
HEK EV	DMSO	Ungated	A	20442	66.14	345.99	372.32	

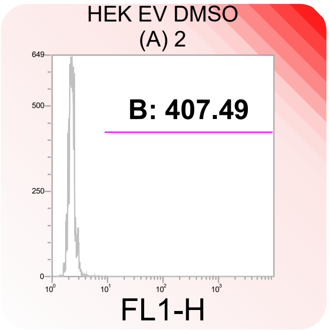

FCS Key 1	FCS Key 2	Gate	Region	Count	%Gated	X Median	X Mean	Error Message
HEK EV	DMSO	A	B	2	0.01	9.14	407.49	

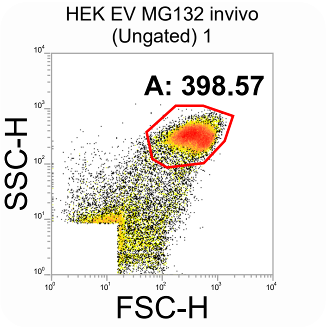

FCS Key 1	FCS Key 2	Gate	Region	Count	%Gated	X Median	X Mean	Error Message
HEK EV	MG132 invivo	Ungated	A	20324	66.00	365.17	398.57	

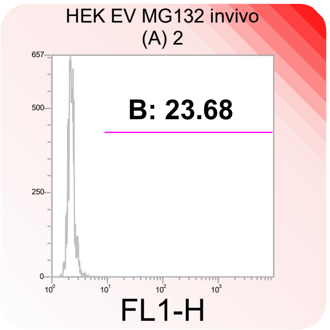

FCS Key 1	FCS Key 2	Gate	Region	Count	%Gated	X Median	X Mean	Error Message
HEK EV	MG132 invivo	A	B	2	0.01	8.82	23.68	

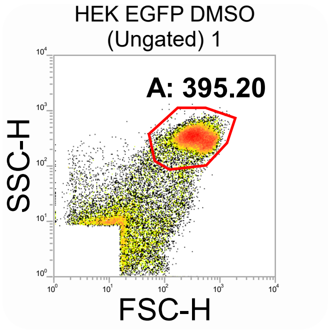

FCS Key 1	FCS Key 2	Gate	Region	Count	%Gated	X Median	X Mean	Error Message
HEK EGFP	DMSO	Ungated	A	20320	60.16	361.90	395.20	

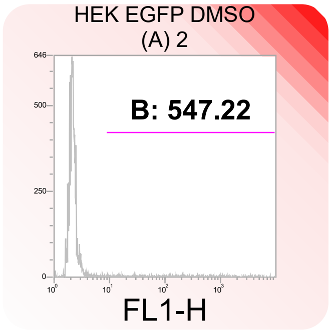

FCS Key 1	FCS Key 2	Gate	Region	Count	%Gated	X Median	X Mean	Error Message
HEK EGFP	DMSO	A	B	621	3.06	105.54	547.22	

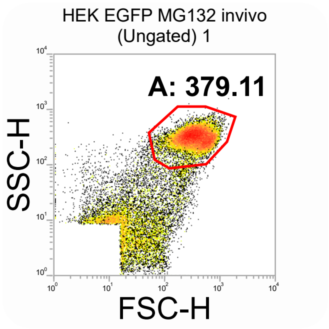

FCS Key 1	FCS Key 2	Gate	Region	Count	%Gated	X Median	X Mean	Error Message
HEK EGFP	MG132 invivo	Ungated	A	20356	63.05	342.89	379.11	

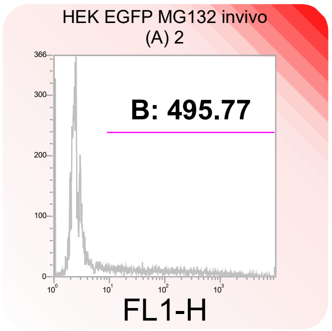

FCS Key 1	FCS Key 2	Gate	Region	Count	%Gated	X Median	X Mean	Error Message
HEK EGFP	MG132 invivo	A	B	5723	28.11	86.60	495.77	

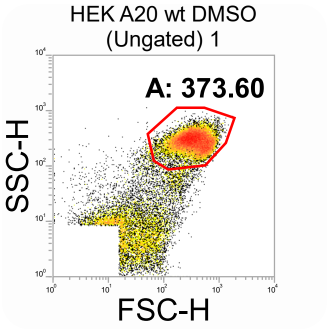

FCS Key 1	FCS Key 2	Gate	Region	Count	%Gated	X Median	X Mean	Error Message
HEK A20 wt	DMSO	Ungated	A	20337	66.03	339.82	373.60	

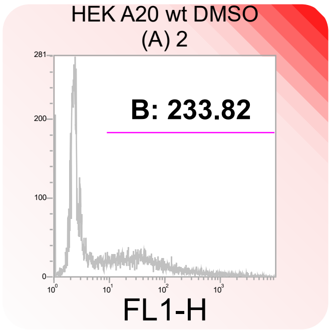

FCS Key 1	FCS Key 2	Gate	Region	Count	%Gated	X Median	X Mean	Error Message
HEK A20 wt	DMSO	A	B	7941	39.05	41.42	233.82	

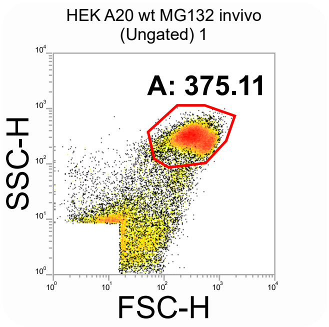

FCS Key 1	FCS Key 2	Gate	Region	Count	%Gated	X Median	X Mean	Error Message
HEK A20 wt	MG132 invivo	Ungated	A	20372	60.69	339.82	375.11	

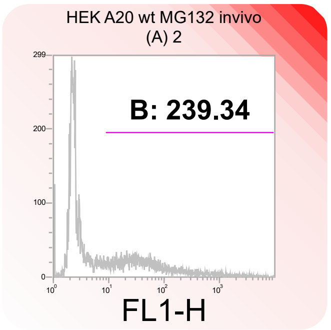

FCS Key 1	FCS Key 2	Gate	Region	Count	%Gated	X Median	X Mean	Error Message
HEK A20 wt	MG132 invivo	A	B	7427	36.46	42.17	239.34	

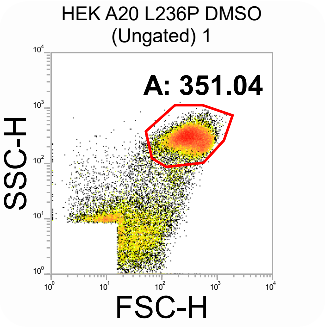

FCS Key 1	FCS Key 2	Gate	Region	Count	%Gated	X Median	X Mean	Error Message
HEK A20 L236P	DMSO	Ungated	A	20413	59.81	316.23	351.04	

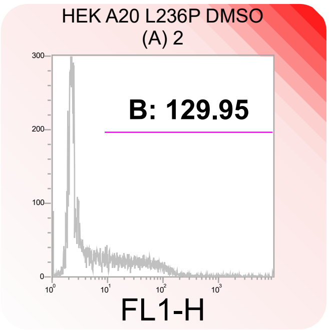

FCS Key 1	FCS Key 2	Gate	Region	Count	%Gated	X Median	X Mean	Error Message
HEK A20 L236P	DMSO	A	B	6363	31.17	33.38	129.95	

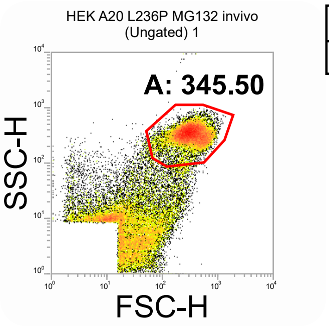

FCS Key 1	FCS Key 2	Gate	Region	Count	%Gated	X Median	X Mean	Error Message
HEK A20 L236P	MG132 invivo	Ungated	A	20511	45.14	313.40	345.50	

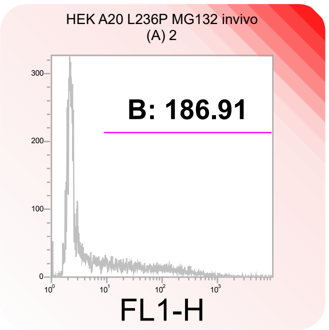

FCS Key 1	FCS Key 2	Gate	Region	Count	%Gated	X Median	X Mean	Error Message
HEK A20 L236P	MG132 invivo	A	B	6381	31.11	47.40	186.91	

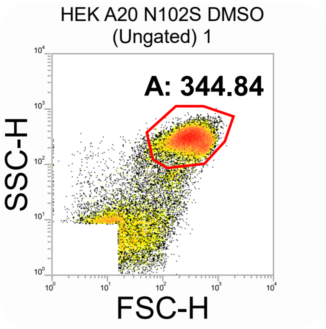

FCS Key 1	FCS Key 2	Gate	Region	Count	%Gated	X Median	X Mean	Error Message
HEK A20 N102S	DMSO	Ungated	A	20608	62.42	313.40	344.84	

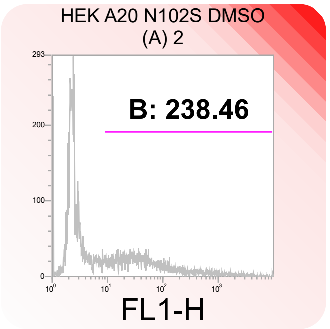

FCS Key 1	FCS Key 2	Gate	Region	Count	%Gated	X Median	X Mean	Error Message
HEK A20 N102S	DMSO	A	B	7971	38.68	42.55	238.46	

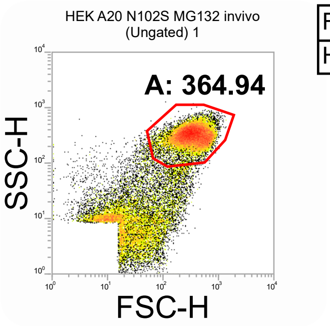

FCS Key 1	FCS Key 2	Gate	Region	Count	%Gated	X Median	X Mean	Error Message
HEK A20 N102S	MG132 invivo	Ungated	A	20422	56.46	330.77	364.94	

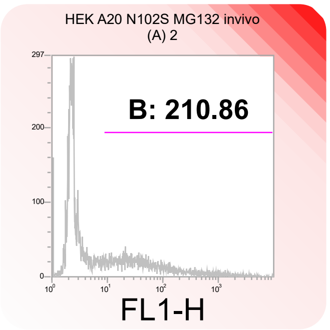

FCS Key 1	FCS Key 2	Gate	Region	Count	%Gated	X Median	X Mean	Error Message
HEK A20 N102S	MG132 invivo	A	B	7292	35.71	40.32	210.86	

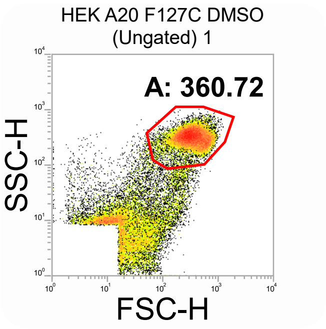

FCS Key 1	FCS Key 2	Gate	Region	Count	%Gated	X Median	X Mean	Error Message
HEK A20 F127C	DMSO	Ungated	A	20437	51.84	324.88	360.72	

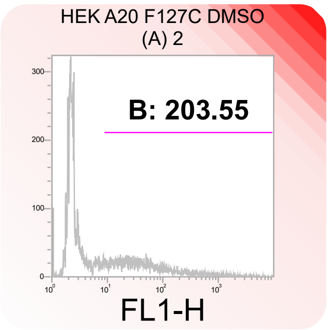

FCS Key 1	FCS Key 2	Gate	Region	Count	%Gated	X Median	X Mean	Error Message
HEK A20 F127C	DMSO	A	B	7042	34.46	39.60	203.55	

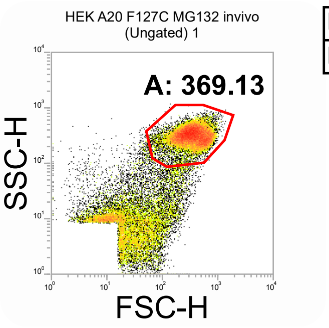

FCS Key 1	FCS Key 2	Gate	Region	Count	%Gated	X Median	X Mean	Error Message
HEK A20 F127C	MG132 invivo	Ungated	A	20502	56.35	336.78	369.13	

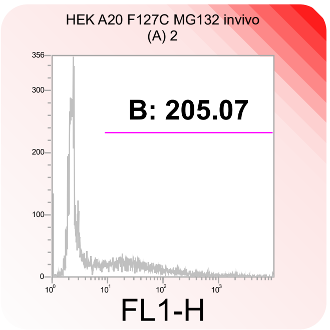

FCS Key 1	FCS Key 2	Gate	Region	Count	%Gated	X Median	X Mean	Error Message
HEK A20 F127C	MG132 invivo	A	B	7064	34.46	38.89	205.07	

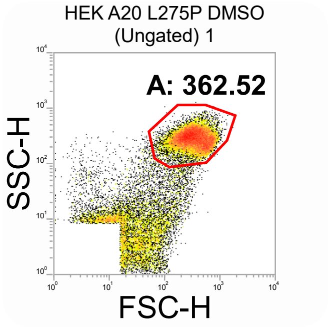

FCS Key 1	FCS Key 2	Gate	Region	Count	%Gated	X Median	X Mean	Error Message
HEK A20 L275P	DMSO	Ungated	A	20291	63.47	327.81	362.52	

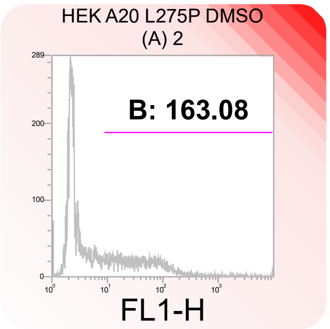

FCS Key 1	FCS Key 2	Gate	Region	Count	%Gated	X Median	X Mean	Error Message
HEK A20 L275P	DMSO	A	B	6944	34.22	38.89	163.08	

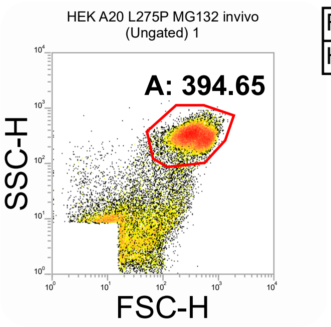

FCS Key 1	FCS Key 2	Gate	Region	Count	%Gated	X Median	X Mean	Error Message
HEK A20 L275P	MG132 invivo	Ungated	A	20233	56.41	361.90	394.65	

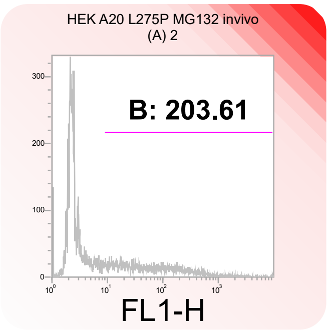

FCS Key 1	FCS Key 2	Gate	Region	Count	%Gated	X Median	X Mean	Error Message
HEK A20 L275P	MG132 invivo	A	B	6811	33.66	55.23	203.61	

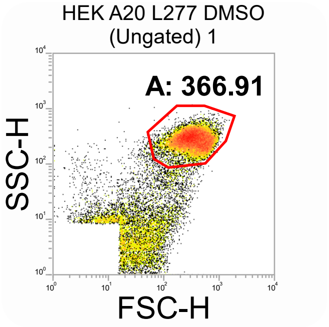

FCS Key 1	FCS Key 2	Gate	Region	Count	%Gated	X Median	X Mean	Error Message
HEK A20 L277	DMSO	Ungated	A	20326	65.97	330.77	366.91	

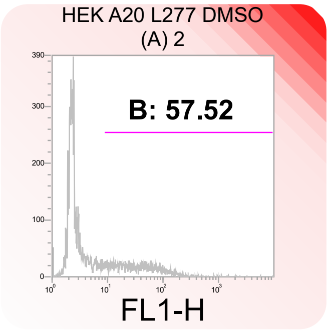

FCS Key 1	FCS Key 2	Gate	Region	Count	%Gated	X Median	X Mean	Error Message
HEK A20 L277	DMSO	A	B	5508	27.10	31.62	57.52	

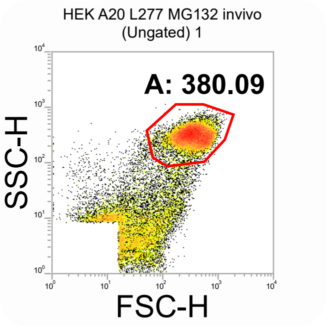

FCS Key 1	FCS Key 2	Gate	Region	Count	%Gated	X Median	X Mean	Error Message
HEK A20 L277	MG132 invivo	Ungated	A	20278	56.96	345.99	380.09	

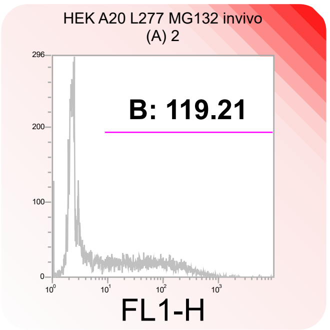

FCS Key 1	FCS Key 2	Gate	Region	Count	%Gated	X Median	X Mean	Error Message
HEK A20 L277	MG132 invivo	A	B	7679	37.87	54.74	119.21	
