# Supplementary material for: A critical region of A20 unveiled by missense TNFAIP3 variations that lead to autoinflammation
Source: eLife. 2023 Jun 21;12:e81280. doi: 10.7554/eLife.81280 (PMC10284599; doi:10.7554/eLife.81280)
Supplement: Figure 4—source data 2. [file elife-81280-fig4-data2.pdf]

Results <Default Table>

FCS Key 1	FCS Key 2	Gate	Region	Count	%Gated	X Median	X Mean	Error Message
HEK NT	no trt	Ungated	A	21062	59.06	327.81	342.47	
HEK NT	no trt	A	B	31	0.15	38.54	127.05	
HEK EV	DMSO	Ungated	A	20277	62.25	381.97	398.62	
HEK EV	DMSO	A	B	20	0.10	38.89	277.70	
HEK EV	MG132 invivo	Ungated	A	20282	55.93	352.27	374.79	
HEK EV	MG132 invivo	A	B	15	0.07	19.63	42.20	
HEK EGFP	DMSO	Ungated	A	20346	65.18	358.66	375.31	
HEK EGFP	DMSO	A	B	9469	46.54	137.00	747.57	
HEK EGFP	MG132 invivo	Ungated	A	20258	57.69	368.47	388.15	
HEK EGFP	MG132 invivo	A	B	8866	43.77	114.44	626.50	
HEK A20 wt	DMSO	Ungated	A	20325	66.35	352.27	374.35	
HEK A20 wt	DMSO	A	B	10400	51.17	46.56	308.52	
HEK A20 wt	MG132 invivo	Ungated	A	20530	54.50	330.77	347.53	
HEK A20 wt	MG132 invivo	A	B	9922	48.33	42.94	292.49	
HEK A20 L236P	DMSO	Ungated	A	20482	66.00	342.89	362.62	
HEK A20 L236P	DMSO	A	B	6114	29.85	31.91	149.91	
HEK A20 L236P	MG132 invivo	Ungated	A	20295	56.05	358.66	385.22	
HEK A20 L236P	MG132 invivo	A	B	6974	34.36	45.32	214.49	
HEK A20 N102S	DMSO	Ungated	A	20359	68.67	345.99	364.06	
HEK A20 N102S	DMSO	A	B	9710	47.69	44.91	299.71	
HEK A20 N102S	MG132 invivo	Ungated	A	20260	52.72	355.45	377.89	
HEK A20 N102S	MG132 invivo	A	B	8913	43.99	42.17	259.96	
HEK A20 F127C	DMSO	Ungated	A	20554	64.79	321.97	343.90	
HEK A20 F127C	DMSO	A	B	9044	44.00	39.60	229.59	
HEK A20 F127C	MG132 invivo	Ungated	A	20449	57.75	355.45	374.06	
HEK A20 F127C	MG132 invivo	A	B	8590	42.01	39.60	253.90	
HEK A20 L275P	DMSO	Ungated	A	20255	66.41	349.12	375.28	
HEK A20 L275P	DMSO	A	B	5584	27.57	28.90	125.93	
HEK A20 L275P	MG132 invivo	Ungated	A	20433	46.78	361.90	392.25	
HEK A20 L275P	MG132 invivo	A	B	6615	32.37	39.60	165.89	
HEK A20 L277	DMSO	Ungated	A	20190	67.99	371.80	399.74	
HEK A20 L277	DMSO	A	B	6432	31.86	31.62	60.19	
HEK A20 L277	MG132 invivo	Ungated	A	20352	39.04	371.80	396.64	
HEK A20 L277	MG132 invivo	A	B	8171	40.15	50.03	115.53	

Overlay 1

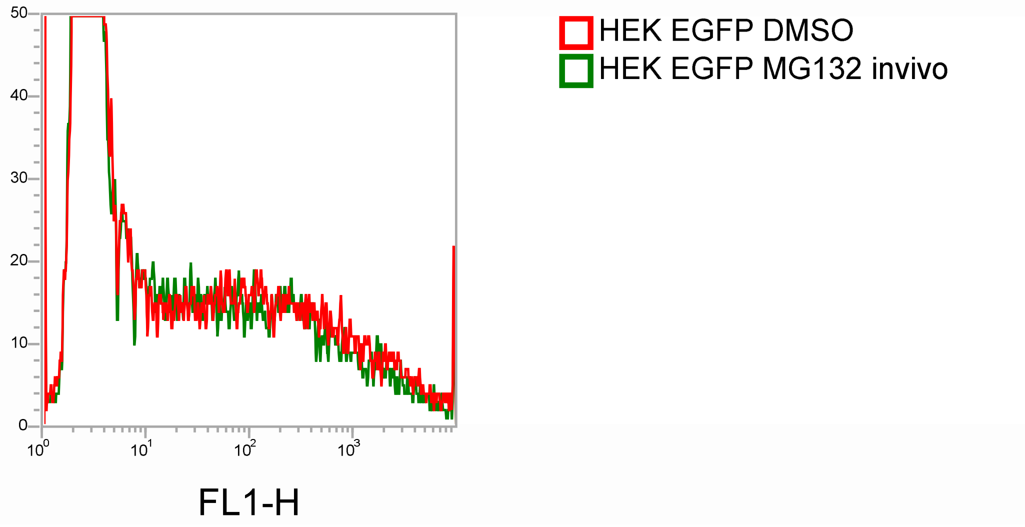

Overlay 2

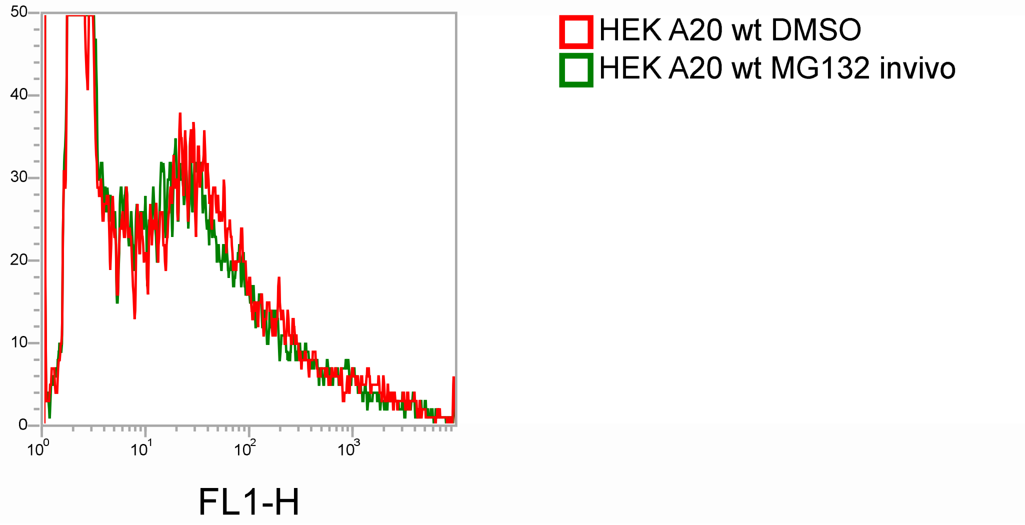

Overlay 3

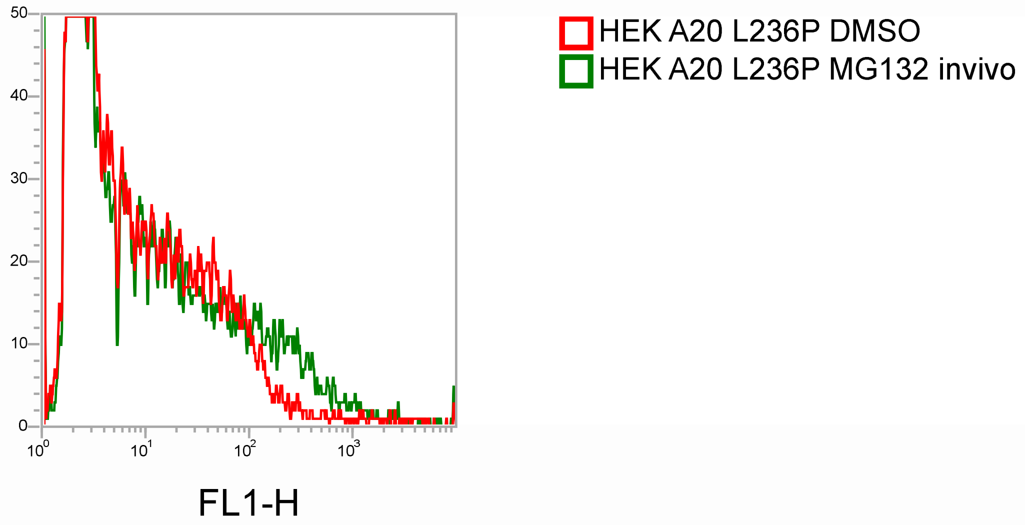

Overlay 4

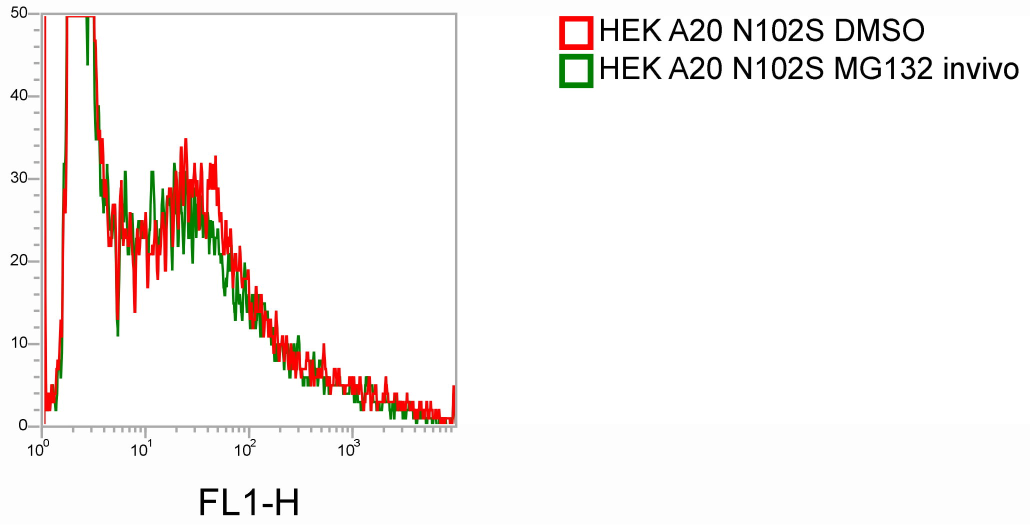

Overlay 5

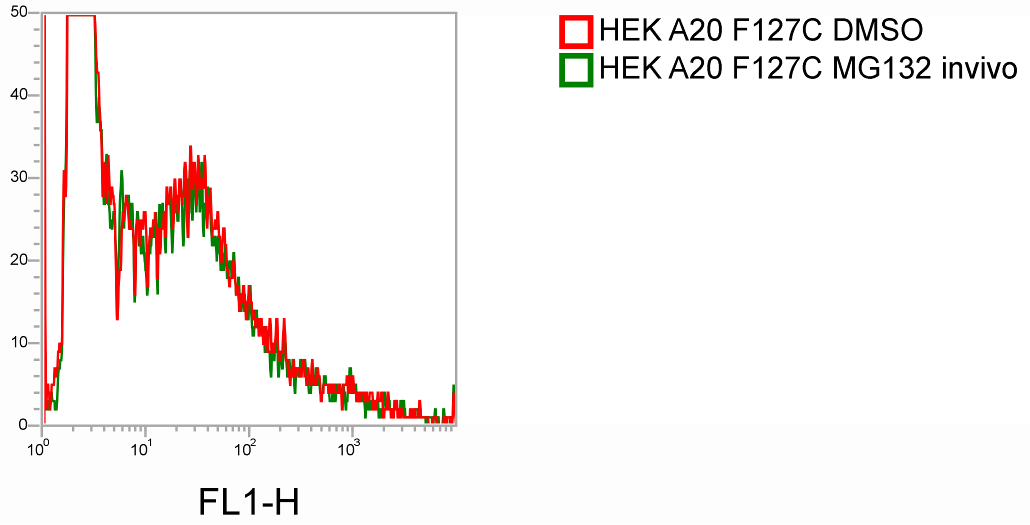

Overlay 6

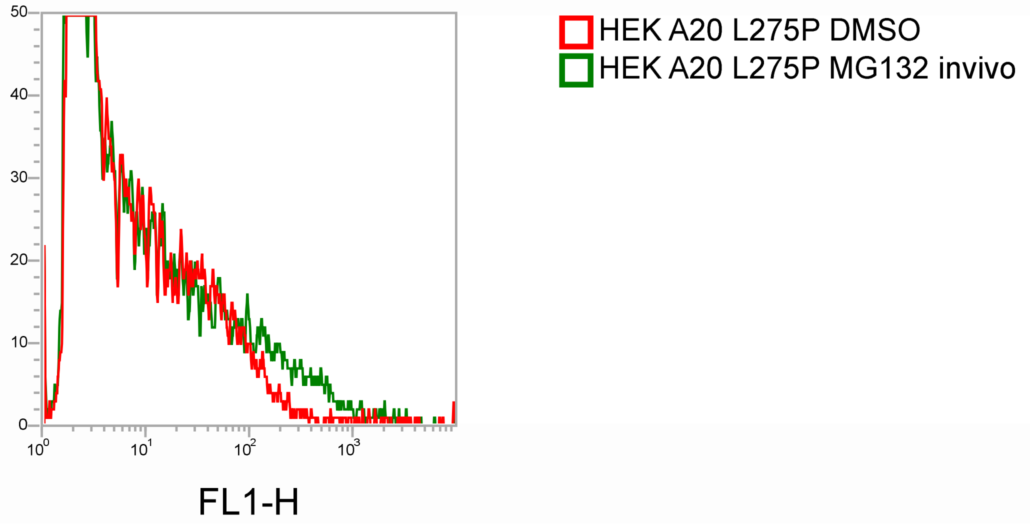

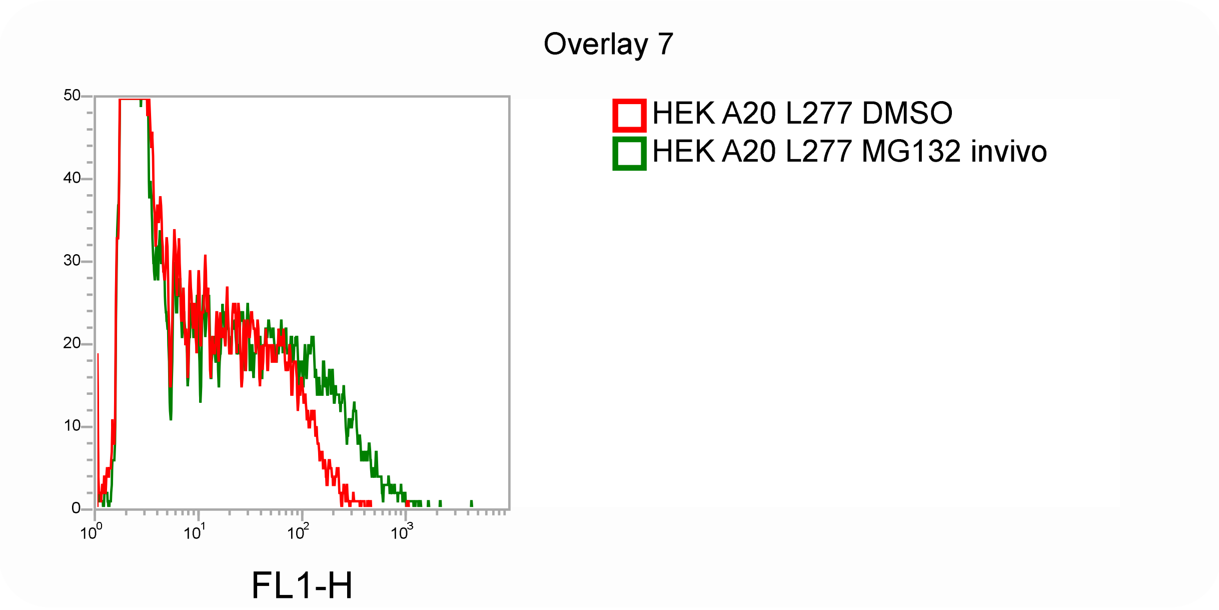

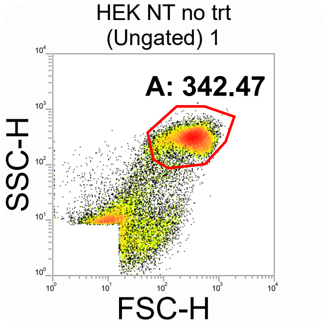

FCS Key 1	FCS Key 2	Gate	Region	Count	%Gated	X Median	X Mean	Error Message
HEK NT	no trt	Ungated	A	21062	59.06	327.81	342.47	

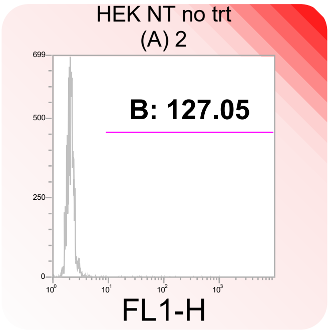

FCS Key 1	FCS Key 2	Gate	Region	Count	%Gated	X Median	X Mean	Error Message
HEK NT	no trt	A	B	31	0.15	38.54	127.05	

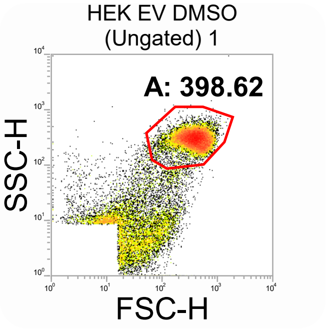

FCS Key 1	FCS Key 2	Gate	Region	Count	%Gated	X Median	X Mean	Error Message
HEK EV	DMSO	Ungated	A	20277	62.25	381.97	398.62	

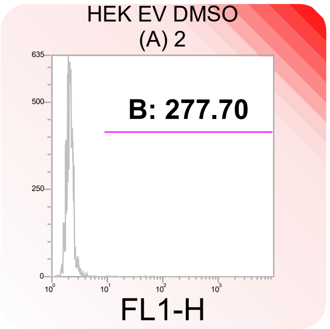

FCS Key 1	FCS Key 2	Gate	Region	Count	%Gated	X Median	X Mean	Error Message
HEK EV	DMSO	A	B	20	0.10	38.89	277.70	

X mean is 277.7 because of 1 cell which is around 900
Checked with lucie
Most likely a cell that jumped from the EGFP well

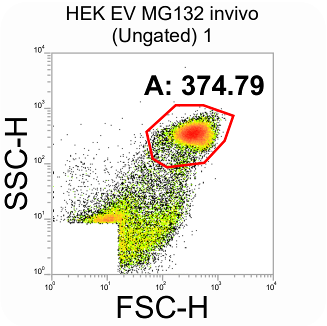

FCS Key 1	FCS Key 2	Gate	Region	Count	%Gated	X Median	X Mean	Error Message
HEK EV	MG132 invivo	Ungated	A	20282	55.93	352.27	374.79	

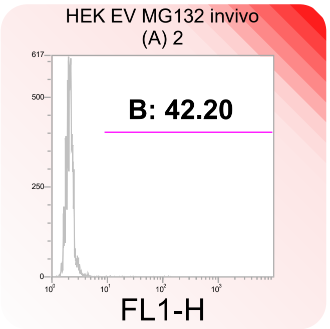

FCS Key 1	FCS Key 2	Gate	Region	Count	%Gated	X Median	X Mean	Error Message
HEK EV	MG132 invivo	A	B	15	0.07	19.63	42.20	

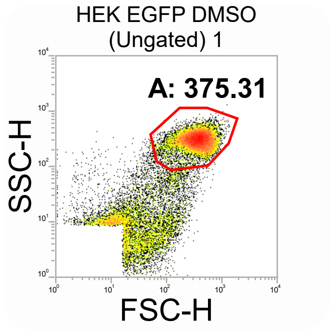

FCS Key 1	FCS Key 2	Gate	Region	Count	%Gated	X Median	X Mean	Error Message
HEK EGFP	DMSO	Ungated	A	20346	65.18	358.66	375.31	

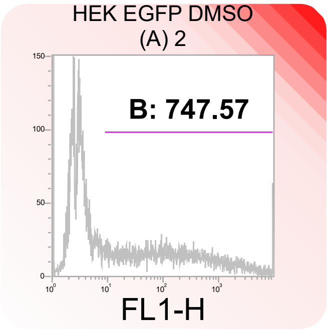

FCS Key 1	FCS Key 2	Gate	Region	Count	%Gated	X Median	X Mean	Error Message
HEK EGFP	DMSO	A	B	9469	46.54	137.00	747.57	

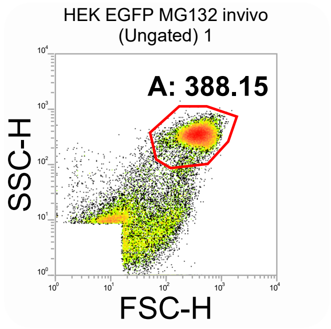

FCS Key 1	FCS Key 2	Gate	Region	Count	%Gated	X Median	X Mean	Error Message
HEK EGFP	MG132 invivo	Ungated	A	20258	57.69	368.47	388.15	

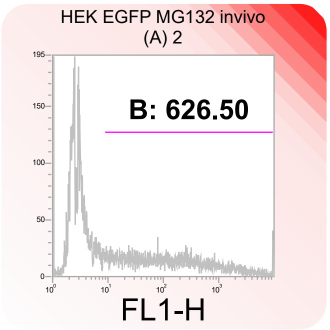

FCS Key 1	FCS Key 2	Gate	Region	Count	%Gated	X Median	X Mean	Error Message
HEK EGFP	MG132 invivo	A	B	8866	43.77	114.44	626.50	

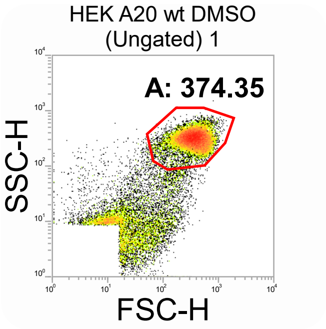

FCS Key 1	FCS Key 2	Gate	Region	Count	%Gated	X Median	X Mean	Error Message
HEK A20 wt	DMSO	Ungated	A	20325	66.35	352.27	374.35	

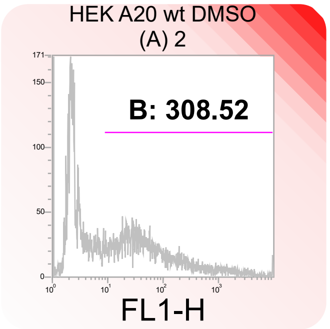

FCS Key 1	FCS Key 2	Gate	Region	Count	%Gated	X Median	X Mean	Error Message
HEK A20 wt	DMSO	A	B	10400	51.17	46.56	308.52	

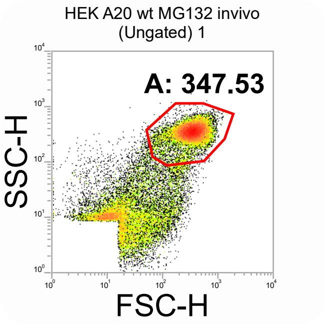

FCS Key 1	FCS Key 2	Gate	Region	Count	%Gated	X Median	X Mean	Error Message
HEK A20 wt	MG132 invivo	Ungated	A	20530	54.50	330.77	347.53	

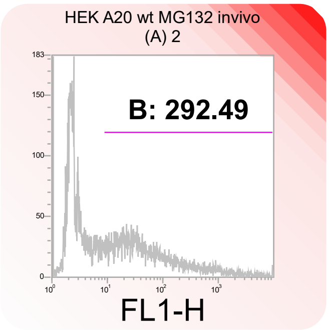

FCS Key 1	FCS Key 2	Gate	Region	Count	%Gated	X Median	X Mean	Error Message
HEK A20 wt	MG132 invivo	A	B	9922	48.33	42.94	292.49	

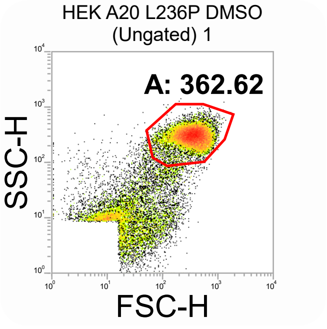

FCS Key 1	FCS Key 2	Gate	Region	Count	%Gated	X Median	X Mean	Error Message
HEK A20 L236P	DMSO	Ungated	A	20482	66.00	342.89	362.62	

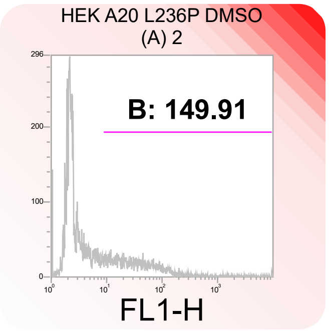

FCS Key 1	FCS Key 2	Gate	Region	Count	%Gated	X Median	X Mean	Error Message
HEK A20 L236P	DMSO	A	B	6114	29.85	31.91	149.91	

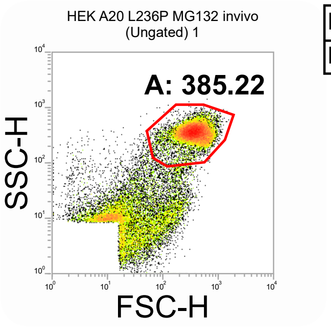

FCS Key 1	FCS Key 2	Gate	Region	Count	%Gated	X Median	X Mean	Error Message
HEK A20 L236P	MG132 invivo	Ungated	A	20295	56.05	358.66	385.22	

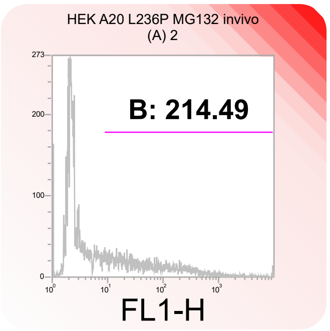

FCS Key 1	FCS Key 2	Gate	Region	Count	%Gated	X Median	X Mean	Error Message
HEK A20 L236P	MG132 invivo	A	B	6974	34.36	45.32	214.49	

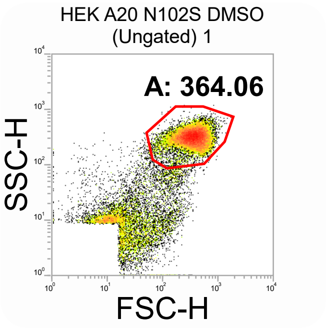

FCS Key 1	FCS Key 2	Gate	Region	Count	%Gated	X Median	X Mean	Error Message
HEK A20 N102S	DMSO	Ungated	A	20359	68.67	345.99	364.06	

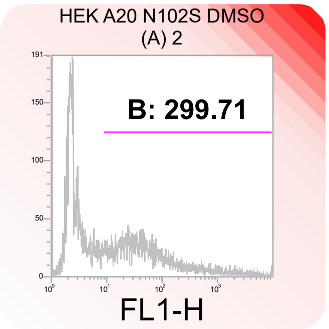

FCS Key 1	FCS Key 2	Gate	Region	Count	%Gated	X Median	X Mean	Error Message
HEK A20 N102S	DMSO	A	B	9710	47.69	44.91	299.71	

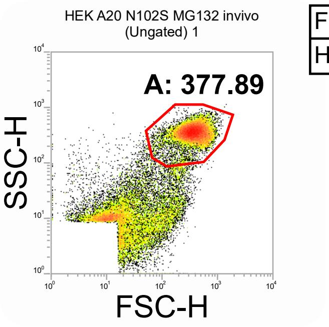

FCS Key 1	FCS Key 2	Gate	Region	Count	%Gated	X Median	X Mean	Error Message
HEK A20 N102S	MG132 invivo	Ungated	A	20260	52.72	355.45	377.89	

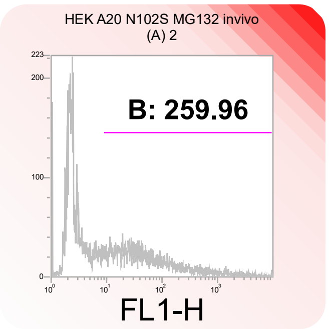

FCS Key 1	FCS Key 2	Gate	Region	Count	%Gated	X Median	X Mean	Error Message
HEK A20 N102S	MG132 invivo	A	B	8913	43.99	42.17	259.96	

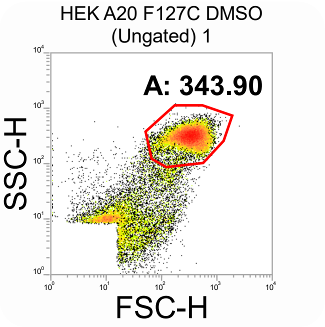

FCS Key 1	FCS Key 2	Gate	Region	Count	%Gated	X Median	X Mean	Error Message
HEK A20 F127C	DMSO	Ungated	A	20554	64.79	321.97	343.90	

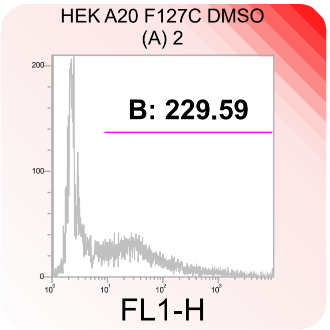

FCS Key 1	FCS Key 2	Gate	Region	Count	%Gated	X Median	X Mean	Error Message
HEK A20 F127C	DMSO	A	B	9044	44.00	39.60	229.59	

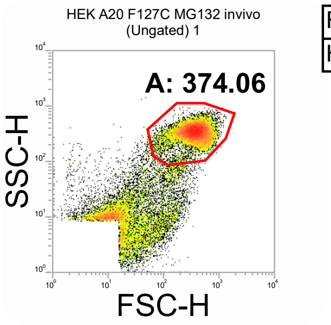

FCS Key 1	FCS Key 2	Gate	Region	Count	%Gated	X Median	X Mean	Error Message
HEK A20 F127C	MG132 invivo	Ungated	A	20449	57.75	355.45	374.06	

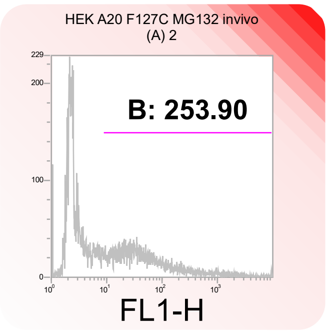

FCS Key 1	FCS Key 2	Gate	Region	Count	%Gated	X Median	X Mean	Error Message
HEK A20 F127C	MG132 invivo	A	B	8590	42.01	39.60	253.90	

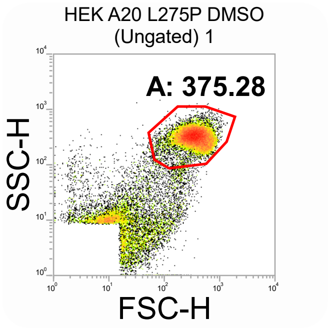

FCS Key 1	FCS Key 2	Gate	Region	Count	%Gated	X Median	X Mean	Error Message
HEK A20 L275P	DMSO	Ungated	A	20255	66.41	349.12	375.28	

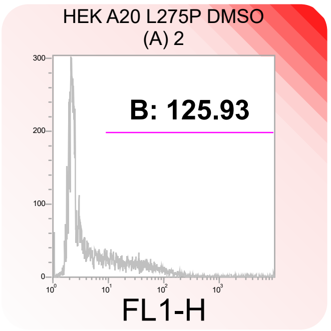

FCS Key 1	FCS Key 2	Gate	Region	Count	%Gated	X Median	X Mean	Error Message
HEK A20 L275P	DMSO	A	B	5584	27.57	28.90	125.93	

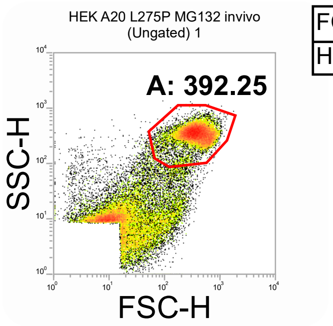

FCS Key 1	FCS Key 2	Gate	Region	Count	%Gated	X Median	X Mean	Error Message
HEK A20 L275P	MG132 invivo	Ungated	A	20433	46.78	361.90	392.25	

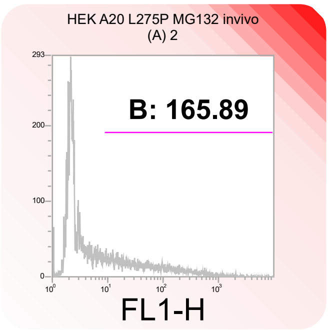

FCS Key 1	FCS Key 2	Gate	Region	Count	%Gated	X Median	X Mean	Error Message
HEK A20 L275P	MG132 invivo	A	B	6615	32.37	39.60	165.89	

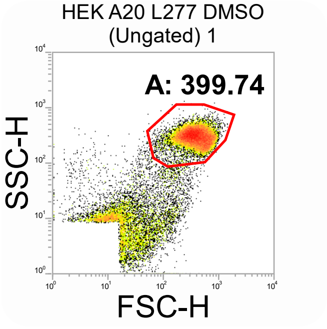

FCS Key 1	FCS Key 2	Gate	Region	Count	%Gated	X Median	X Mean	Error Message
HEK A20 L277	DMSO	Ungated	A	20190	67.99	371.80	399.74	

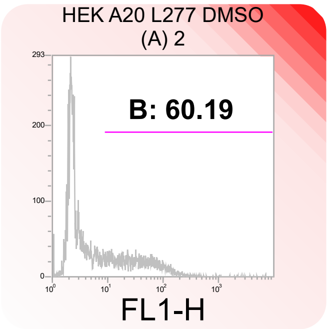

FCS Key 1	FCS Key 2	Gate	Region	Count	%Gated	X Median	X Mean	Error Message
HEK A20 L277	DMSO	A	B	6432	31.86	31.62	60.19	

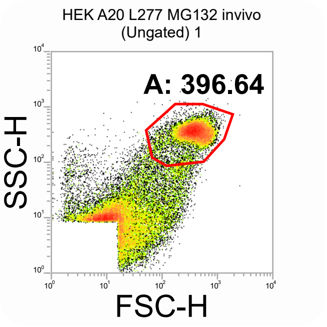

FCS Key 1	FCS Key 2	Gate	Region	Count	%Gated	X Median	X Mean	Error Message
HEK A20 L277	MG132 invivo	Ungated	A	20352	39.04	371.80	396.64	

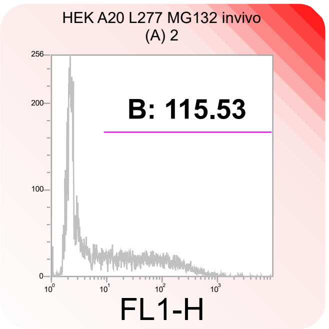

FCS Key 1	FCS Key 2	Gate	Region	Count	%Gated	X Median	X Mean	Error Message
HEK A20 L277	MG132 invivo	A	B	8171	40.15	50.03	115.53	
