# Supplementary material for: A critical region of A20 unveiled by missense TNFAIP3 variations that lead to autoinflammation
Source: eLife. 2023 Jun 21;12:e81280. doi: 10.7554/eLife.81280 (PMC10284599; doi:10.7554/eLife.81280)
Supplement: Figure 4—source data 3. [file elife-81280-fig4-data3.pdf]

Results <Default Table>

FCS Key 1	FCS Key 2	Gate	Region	Count	%Gated	X Median	X Mean	Error Message
HEK NT	no trt	Ungated	A	20405	67.03	352.27	367.27	
HEK NT	no trt	A	B	0	0.00	ERROR	ERROR	
HEK EV	DMSO	Ungated	A	20345	67.10	358.66	373.04	
HEK EV	DMSO	A	B	3	0.01	47.83	39.70	
HEK EV	MG132 invivo	Ungated	A	20231	56.98	375.16	394.33	
HEK EV	MG132 invivo	A	B	0	0.00	ERROR	ERROR	
HEK EGFP	DMSO	Ungated	A	20236	66.69	381.97	397.50	
HEK EGFP	DMSO	A	B	6087	30.08	127.49	682.88	
HEK EGFP	MG132 invivo	Ungated	A	20264	57.86	388.91	408.50	
HEK EGFP	MG132 invivo	A	B	5651	27.89	106.50	636.63	
HEK A20 wt	DMSO	Ungated	A	20162	68.33	378.55	397.79	
HEK A20 wt	DMSO	A	B	5568	27.62	35.55	218.81	
HEK A20 wt	MG132 invivo	Ungated	A	20192	59.55	399.54	419.06	
HEK A20 wt	MG132 invivo	A	B	5651	27.99	34.29	198.08	
HEK A20 L236P	DMSO	Ungated	A	20496	61.34	378.55	390.55	
HEK A20 L236P	DMSO	A	B	4784	23.34	30.23	128.39	
HEK A20 L236P	MG132 invivo	Ungated	A	20449	53.46	365.17	382.80	
HEK A20 L236P	MG132 invivo	A	B	5083	24.86	41.05	174.51	
HEK A20 N102S	DMSO	Ungated	A	20564	59.21	375.16	388.64	
HEK A20 N102S	DMSO	A	B	6169	30.00	40.32	214.90	
HEK A20 N102S	MG132 invivo	Ungated	A	20386	70.16	355.45	372.58	
HEK A20 N102S	MG132 invivo	A	B	6753	33.13	36.19	206.81	
HEK A20 F127C	DMSO	Ungated	A	20349	62.53	392.42	408.04	
HEK A20 F127C	DMSO	A	B	6802	33.43	37.18	214.24	
HEK A20 F127C	MG132 invivo	Ungated	A	20376	51.06	365.17	383.16	
HEK A20 F127C	MG132 invivo	A	B	5295	25.99	32.20	168.24	
HEK A20 L275P	DMSO	Ungated	A	20384	62.52	375.16	389.52	
HEK A20 L275P	DMSO	A	B	4825	23.67	31.34	124.67	
HEK A20 L275P	MG132 invivo	Ungated	A	20396	52.09	365.17	385.22	
HEK A20 L275P	MG132 invivo	A	B	5311	26.04	46.14	185.34	
HEK A20 L277	DMSO	Ungated	A	20372	70.75	352.27	379.45	
HEK A20 L277	DMSO	A	B	5074	24.91	32.20	60.49	
HEK A20 L277	MG132 invivo	Ungated	A	20296	60.63	395.96	422.78	
HEK A20 L277	MG132 invivo	A	B	5758	28.37	45.73	106.36	

Overlay 1

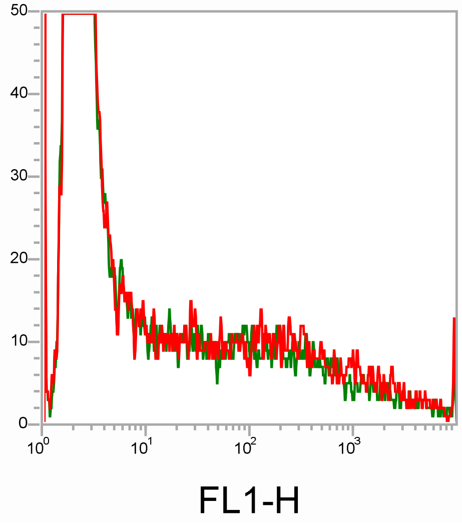

HEK EGFP DMSO
HEK EGFP MG132 invivo

Overlay 2

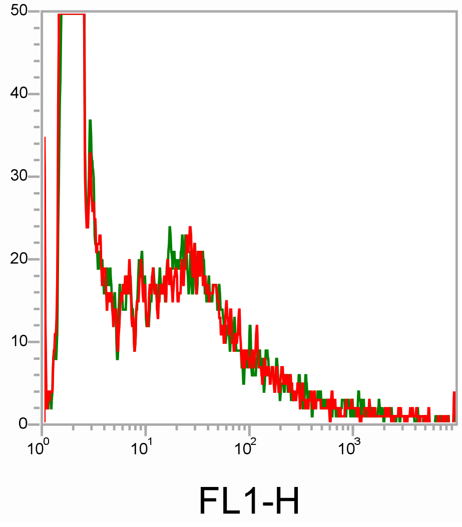

HEK A20 wt DMSO
HEK A20 wt MG132 invivo

Overlay 3

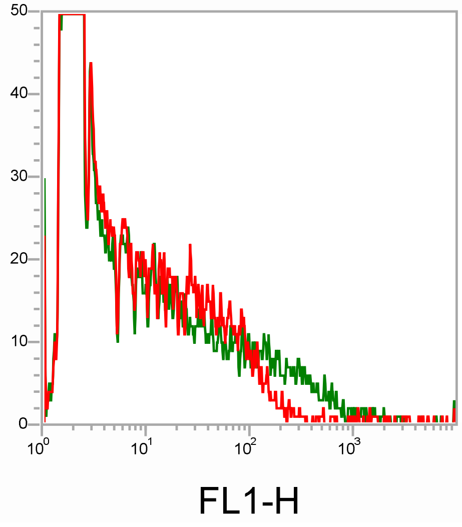

HEK A20 L236P DMSO
HEK A20 L236P MG132 invivo

Overlay 4

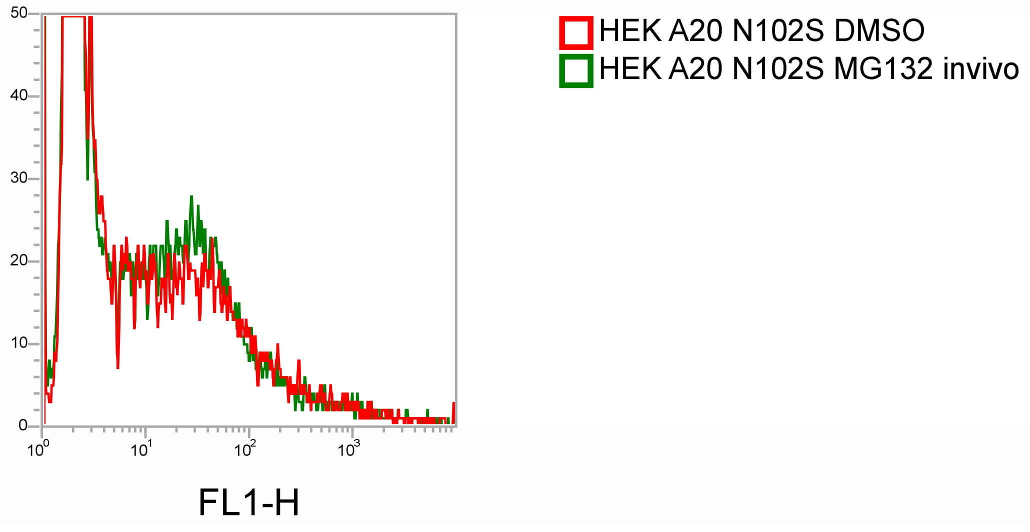

Overlay 5

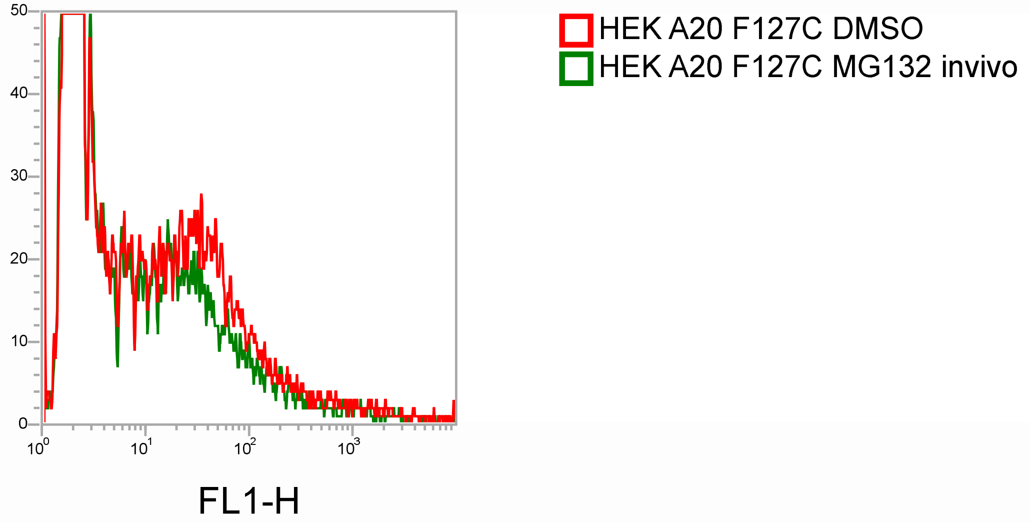

Overlay 6

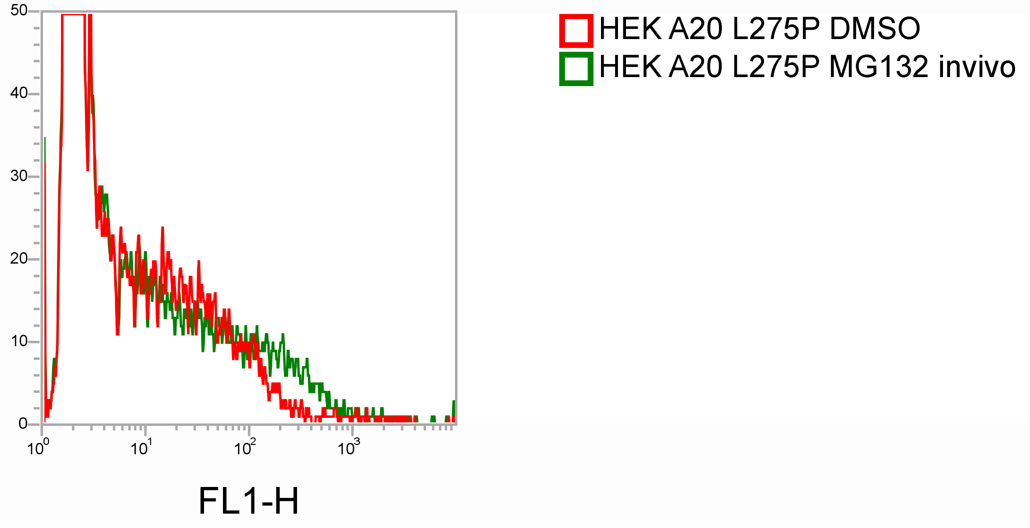

Overlay 7

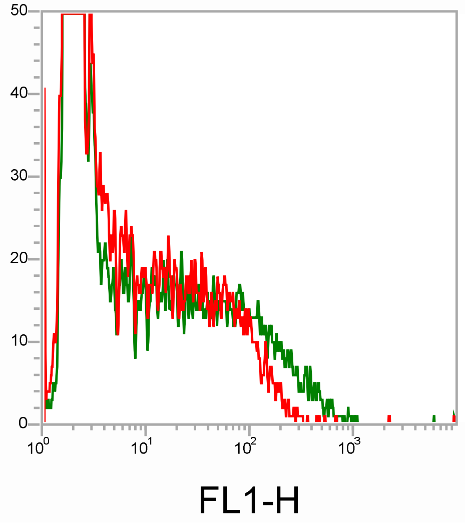

HEK NT no trt
(Ungated) 1

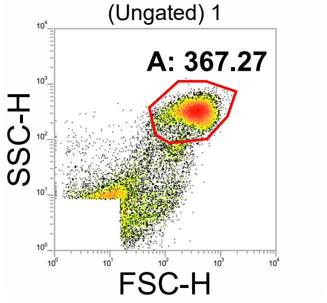

FCS Key 1	FCS Key 2	Gate	Region	Count	%Gated	X Median	X Mean	Error Message
HEK NT	no trt	Ungated	A	20405	67.03	352.27	367.27	

HEK NT no trt
(A) 2

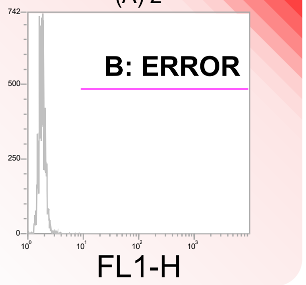

FCS Key 1	FCS Key 2	Gate	Region	Count	%Gated	X Median	X Mean	Error Message
HEK NT	no trt	A	B	0	0.00	ERROR	ERROR	

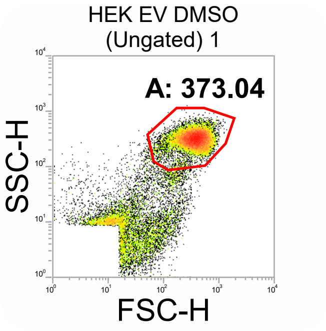

FCS Key 1	FCS Key 2	Gate	Region	Count	%Gated	X Median	X Mean	Error Message
HEK EV	DMSO	Ungated	A	20345	67.10	358.66	373.04	

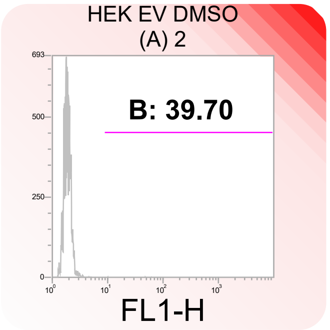

FCS Key 1	FCS Key 2	Gate	Region	Count	%Gated	X Median	X Mean	Error Message
HEK EV	DMSO	A	B	3	0.01	47.83	39.70	

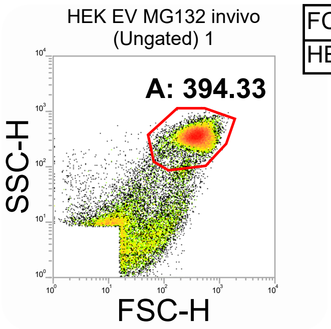

FCS Key 1	FCS Key 2	Gate	Region	Count	%Gated	X Median	X Mean	Error Message
HEK EV	MG132 invivo	Ungated	A	20231	56.98	375.16	394.33	

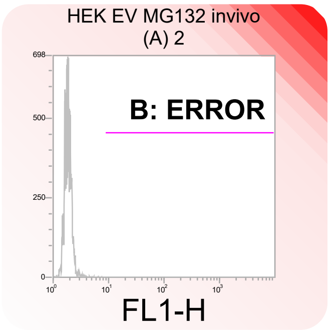

FCS Key 1	FCS Key 2	Gate	Region	Count	%Gated	X Median	X Mean	Error Message
HEK EV	MG132 invivo	A	B	0	0.00	ERROR	ERROR	

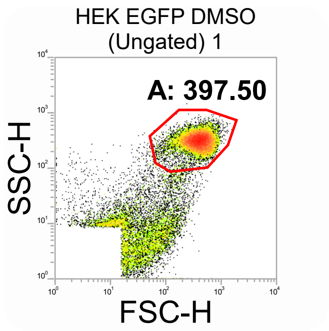

FCS Key 1	FCS Key 2	Gate	Region	Count	%Gated	X Median	X Mean	Error Message
HEK EGFP	DMSO	Ungated	A	20236	66.69	381.97	397.50	

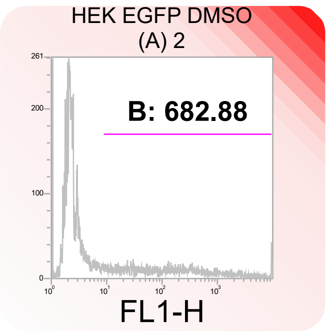

FCS Key 1	FCS Key 2	Gate	Region	Count	%Gated	X Median	X Mean	Error Message
HEK EGFP	DMSO	A	B	6087	30.08	127.49	682.88	

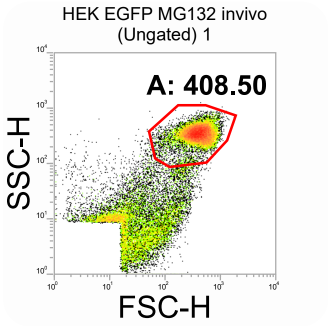

FCS Key 1	FCS Key 2	Gate	Region	Count	%Gated	X Median	X Mean	Error Message
HEK EGFP	MG132 invivo	Ungated	A	20264	57.86	388.91	408.50	

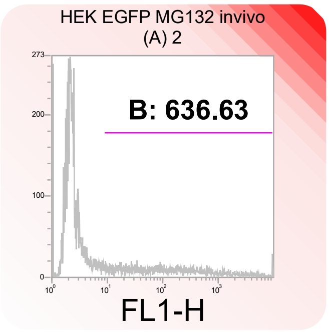

FCS Key 1	FCS Key 2	Gate	Region	Count	%Gated	X Median	X Mean	Error Message
HEK EGFP	MG132 invivo	A	B	5651	27.89	106.50	636.63	

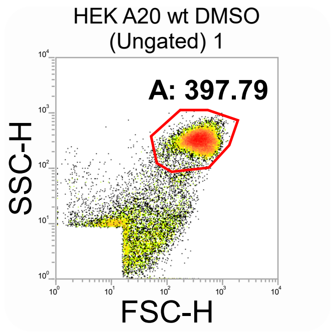

FCS Key 1	FCS Key 2	Gate	Region	Count	%Gated	X Median	X Mean	Error Message
HEK A20 wt	DMSO	Ungated	A	20162	68.33	378.55	397.79	

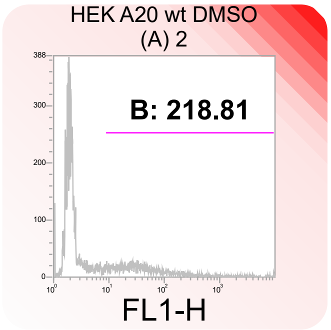

FCS Key 1	FCS Key 2	Gate	Region	Count	%Gated	X Median	X Mean	Error Message
HEK A20 wt	DMSO	A	B	5568	27.62	35.55	218.81	

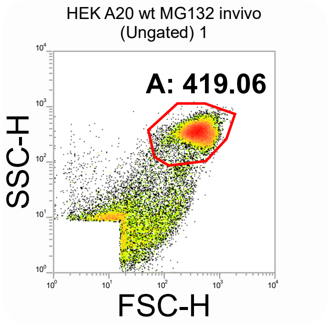

FCS Key 1	FCS Key 2	Gate	Region	Count	%Gated	X Median	X Mean	Error Message
HEK A20 wt	MG132 invivo	Ungated	A	20192	59.55	399.54	419.06	

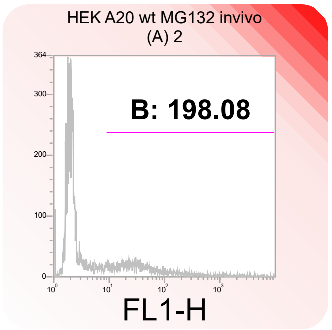

FCS Key 1	FCS Key 2	Gate	Region	Count	%Gated	X Median	X Mean	Error Message
HEK A20 wt	MG132 invivo	A	B	5651	27.99	34.29	198.08	

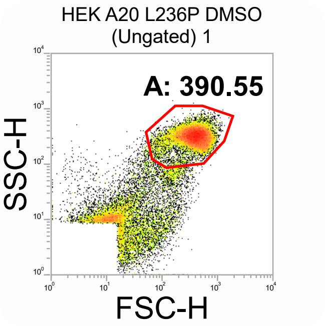

FCS Key 1	FCS Key 2	Gate	Region	Count	%Gated	X Median	X Mean	Error Message
HEK A20 L236P	DMSO	Ungated	A	20496	61.34	378.55	390.55	

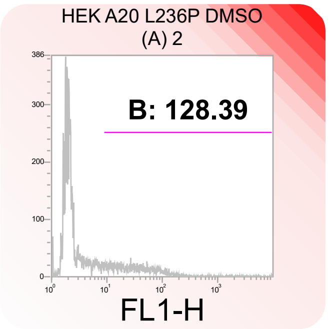

FCS Key 1	FCS Key 2	Gate	Region	Count	%Gated	X Median	X Mean	Error Message
HEK A20 L236P	DMSO	A	B	4784	23.34	30.23	128.39	

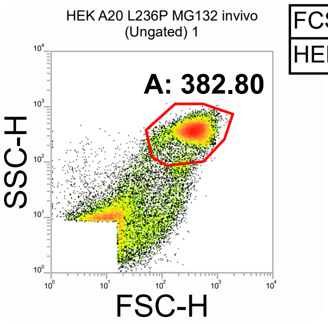

FCS Key 1	FCS Key 2	Gate	Region	Count	%Gated	X Median	X Mean	Error Message
HEK A20 L236P	MG132 invivo	Ungated	A	20449	53.46	365.17	382.80	

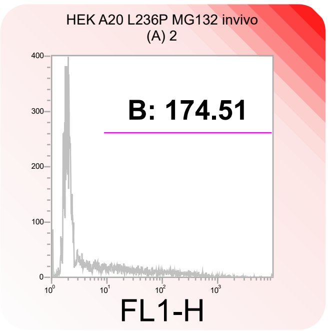

FCS Key 1	FCS Key 2	Gate	Region	Count	%Gated	X Median	X Mean	Error Message
HEK A20 L236P	MG132 invivo	A	B	5083	24.86	41.05	174.51	

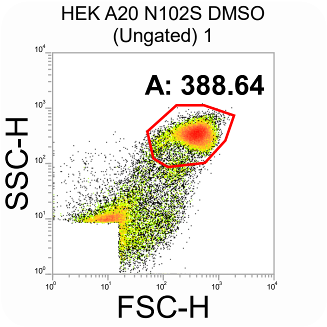

FCS Key 1	FCS Key 2	Gate	Region	Count	%Gated	X Median	X Mean	Error Message
HEK A20 N102S	DMSO	Ungated	A	20564	59.21	375.16	388.64	

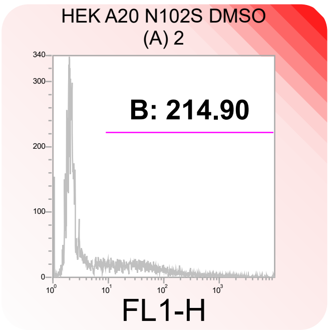

FCS Key 1	FCS Key 2	Gate	Region	Count	%Gated	X Median	X Mean	Error Message
HEK A20 N102S	DMSO	A	B	6169	30.00	40.32	214.90	

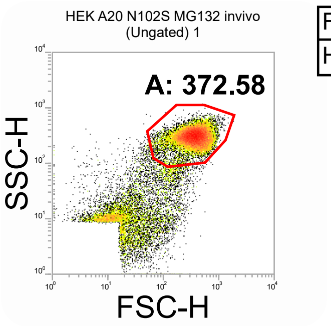

FCS Key 1	FCS Key 2	Gate	Region	Count	%Gated	X Median	X Mean	Error Message
HEK A20 N102S	MG132 invivo	Ungated	A	20386	70.16	355.45	372.58	

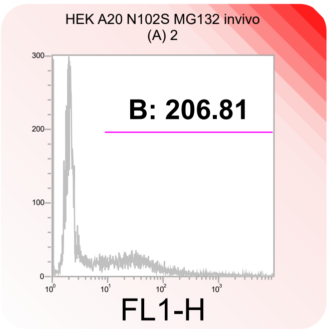

FCS Key 1	FCS Key 2	Gate	Region	Count	%Gated	X Median	X Mean	Error Message
HEK A20 N102S	MG132 invivo	A	B	6753	33.13	36.19	206.81	

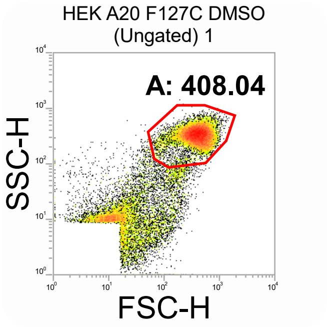

FCS Key 1	FCS Key 2	Gate	Region	Count	%Gated	X Median	X Mean	Error Message
HEK A20 F127C	DMSO	Ungated	A	20349	62.53	392.42	408.04	

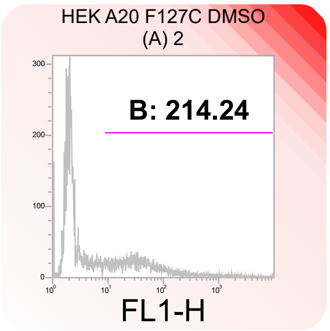

FCS Key 1	FCS Key 2	Gate	Region	Count	%Gated	X Median	X Mean	Error Message
HEK A20 F127C	DMSO	A	B	6802	33.43	37.18	214.24	

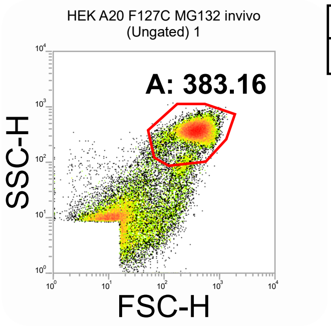

FCS Key 1	FCS Key 2	Gate	Region	Count	%Gated	X Median	X Mean	Error Message
HEK A20 F127C	MG132 invivo	Ungated	A	20376	51.06	365.17	383.16	

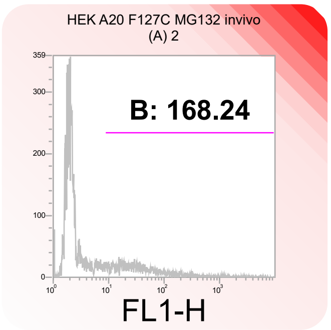

FCS Key 1	FCS Key 2	Gate	Region	Count	%Gated	X Median	X Mean	Error Message
HEK A20 F127C	MG132 invivo	A	B	5295	25.99	32.20	168.24	

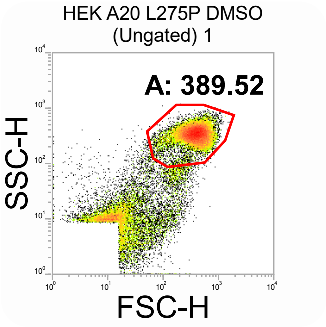

FCS Key 1	FCS Key 2	Gate	Region	Count	%Gated	X Median	X Mean	Error Message
HEK A20 L275P	DMSO	Ungated	A	20384	62.52	375.16	389.52	

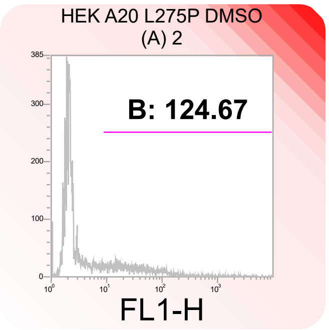

FCS Key 1	FCS Key 2	Gate	Region	Count	%Gated	X Median	X Mean	Error Message
HEK A20 L275P	DMSO	A	B	4825	23.67	31.34	124.67	

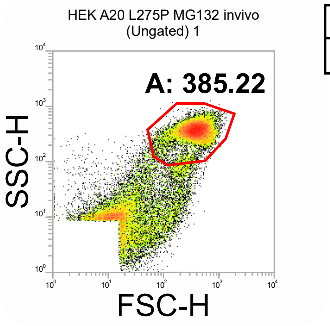

FCS Key 1	FCS Key 2	Gate	Region	Count	%Gated	X Median	X Mean	Error Message
HEK A20 L275P	MG132 invivo	Ungated	A	20396	52.09	365.17	385.22	

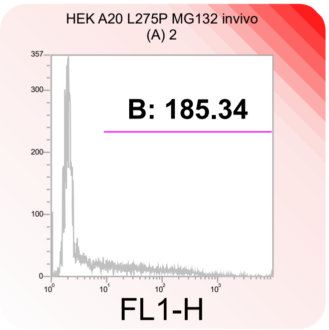

FCS Key 1	FCS Key 2	Gate	Region	Count	%Gated	X Median	X Mean	Error Message
HEK A20 L275P	MG132 invivo	A	B	5311	26.04	46.14	185.34	

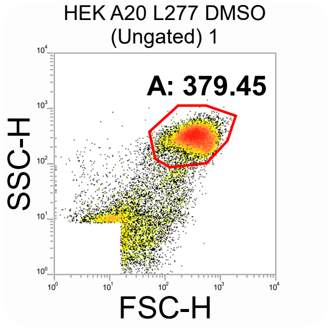

FCS Key 1	FCS Key 2	Gate	Region	Count	%Gated	X Median	X Mean	Error Message
HEK A20 L277	DMSO	Ungated	A	20372	70.75	352.27	379.45	

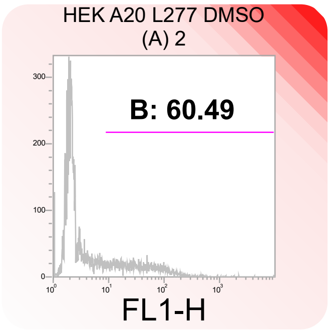

FCS Key 1	FCS Key 2	Gate	Region	Count	%Gated	X Median	X Mean	Error Message
HEK A20 L277	DMSO	A	B	5074	24.91	32.20	60.49	

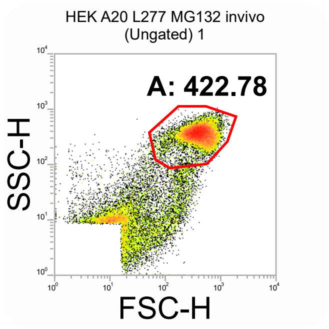

FCS Key 1	FCS Key 2	Gate	Region	Count	%Gated	X Median	X Mean	Error Message
HEK A20 L277	MG132 invivo	Ungated	A	20296	60.63	395.96	422.78	

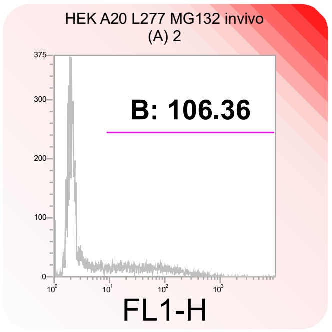

FCS Key 1	FCS Key 2	Gate	Region	Count	%Gated	X Median	X Mean	Error Message
HEK A20 L277	MG132 invivo	A	B	5758	28.37	45.73	106.36	
